# ^18^F-AzaFol for Detection of Folate Receptor-β Positive Macrophages in Experimental Interstitial Lung Disease—A Proof-of-Concept Study

**DOI:** 10.3389/fimmu.2019.02724

**Published:** 2019-11-22

**Authors:** Janine Schniering, Martina Benešová, Matthias Brunner, Stephanie Haller, Susan Cohrs, Thomas Frauenfelder, Bart Vrugt, Carol Feghali-Bostwick, Roger Schibli, Oliver Distler, Cristina Müller, Britta Maurer

**Affiliations:** ^1^Department of Rheumatology, Center of Experimental Rheumatology, University Hospital Zurich, Zurich, Switzerland; ^2^Center for Radiopharmaceutical Sciences, Paul Scherrer Institute, Villigen, Switzerland; ^3^Department of Chemistry and Applied Biosciences, ETH Zurich, Zurich, Switzerland; ^4^Institute of Diagnostic and Interventional Radiology, University Hospital Zurich, Zurich, Switzerland; ^5^Institute of Pathology and Molecular Pathology, University Hospital Zurich, Zurich, Switzerland; ^6^Division of Rheumatology & Immunology, Medical University of South Carolina, Charleston, SC, United States

**Keywords:** interstitial lung disease, imaging biomarkers, animal model of lung fibrosis, macrophages, folate receptor, positron emission tomography, inflammation, folate-based ^18^F-PET tracer

## Abstract

**Background:** Interstitial lung disease (ILD) is a common and severe complication in rheumatic diseases. Folate receptor-β is expressed on activated, but not resting macrophages which play a key role in dysregulated tissue repair including ILD. We therefore aimed to pre-clinically evaluate the potential of ^18^F-AzaFol-based PET/CT (positron emission computed tomography/computed tomography) for the specific detection of macrophage-driven pathophysiologic processes in experimental ILD.

**Methods:** The pulmonary expression of folate receptor-β was analyzed in patients with different subtypes of ILD as well as in bleomycin (BLM)-treated mice and respective controls using immunohistochemistry. PET/CT was performed at days 3, 7, and 14 after BLM instillation using the ^18^F-based folate radiotracer ^18^F-AzaFol. The specific pulmonary accumulation of the radiotracer was assessed by *ex vivo* PET/CT scans and quantified by *ex vivo* biodistribution studies.

**Results:** Folate receptor-β expression was 3- to 4-fold increased in patients with fibrotic ILD, including idiopathic pulmonary fibrosis and connective tissue disease-related ILD, and significantly correlated with the degree of lung remodeling. A similar increase in the expression of folate receptor-β was observed in experimental lung fibrosis, where it also correlated with disease extent. In the mouse model of BLM-induced ILD, pulmonary accumulation of ^18^F-AzaFol reflected macrophage-related disease development with good correlation of folate receptor-β positivity with radiotracer uptake. In the *ex vivo* imaging and biodistribution studies, the maximum lung accumulation was observed at day 7 with a mean accumulation of 1.01 ± 0.30% injected activity/lung in BLM-treated vs. control animals (0.31 ± 0.06% % injected activity/lung; *p* < 0.01).

**Conclusion:** Our preclinical proof-of-concept study demonstrated the potential of ^18^F-AzaFol as a novel imaging tool for the visualization of macrophage-driven fibrotic lung diseases.

## Introduction

In the US, 45% of deaths can be attributed to fibrotic disorders including pulmonary fibrosis (1), for which a global rise in mortality is observed (2). This large and heterogeneous group of parenchymal lung disorders, termed interstitial lung disease (ILD) shares the common feature of pulmonary fibrosis resulting in impaired respiratory function and often failure. The most prevalent forms of ILD are idiopathic pulmonary fibrosis (IPF) and ILD associated with connective tissue diseases (CTD-ILD). CTDs commonly complicated by ILD include systemic sclerosis (SSc) (3), idiopathic inflammatory myopathies (4), rheumatoid arthritis (5), systemic lupus erythematous (6), Sjögren's syndrome (7), mixed connective tissue disease (8), and undifferentiated connective tissue disease (9). Among the different CTD-ILDs, ILD is most prevalent in SSc with 70–90% of SSc patients developing ILD (10). The life expectancy is markedly reduced, especially in IPF and SSc-ILD, with a median survival of 2–3 years from diagnosis (2, 10).

Despite their clinical heterogeneity, increasing data suggest that in ILD fibrosis develops due to the same dysregulation of wound-healing mechanisms (11, 12).Whereas cell death of alveolar epithelial cells is considered the key trigger of ILD (13, 14), a growing body of (pre-)clinical data point to a similarly crucial pathogenic role of pulmonary macrophages and macrophage-released factors (15, 16). Macrophage activation was shown throughout different stages of ILD including early/mild (17), intermediate (18) as well as end-stage/severe stages (19), and also in different ILD etiologies (20). These observations argue either for a persistent role of macrophages throughout the disease process or for the existence of “inflammatory” or “macrophage-driven” subtypes of ILD (21, 22). Importantly, the persistence of macrophages seems to correlate with poor prognosis and reduced overall survival (17, 23). Thus, the development of macrophage-targeted imaging techniques for prognostic and treatment purposes in ILD might represent a valuable approach to improve the deleterious disease outcome (15).

Molecular imaging, including nuclear imaging approaches such as positron emission tomography (PET) are sensitive and allow the non-invasive visualization of pathophysiologic processes in real-time. This is a unique advantage over conventional morphological imaging modalities such as high resolution computed tomography (HRCT) scans or magnetic resonance imaging (MRI) (24). These conventional imaging techniques depict anatomical changes in organ architecture with high spatial resolution. They can, however, neither provide information on whether the observed changes are signs of inactive or active tissue remodeling, nor discriminate inflammatory from fibrotic processes, a crucial information for informed clinical decision making (12, 25). An example is the presence of ground glass opacities, which commonly are considered to reflect alveolitis. However, the notion of alveolitis being synonymous to inflammation has been abandoned in fibrosing ILD, since early fibrotic interstitial changes have the same appearance on HRCT (26).

In recent years, several studies have investigated the potential of 2-deoxy-2-[^18^F]fluoro-D-glucose [^18^F]FDG-PET/CT for diagnosis of ILD. [^18^F]FDG is an unspecific, metabolic radiotracer for the assessment of cellular glucose metabolism, which has been shown to be elevated in ILD (27, 28). A disadvantage for its use in diagnosis and monitoring of ILD is that [^18^F]FDG signals reflect metabolic activity, which can arise from both inflammatory and fibrotic cell types and can occur during different disease stages including those of stabilization or repair. This lacking discrimination of pathophysiologic stages of ILD diminishes the value of [^18^F]FDG-PET/CT for informed treatment decisions and monitoring of therapeutic responses (29, 30).

In contrast, imaging approaches using target-specific radiotracers ideally aiming at a single key cell type in ILD may overcome this limitation of [^18^F]FDG-PET/CT. So far, only few approaches have been successfully applied pre-clinically in ILD (31–36).

Activated macrophages express folate receptor-β (FR-β) in various pathological conditions including cancer and inflammatory diseases (37–39), whereas the number of FR-β-expressing macrophages is very low under physiological conditions (37, 40).

FR-β is a glycosylphosphatidylinositol (GPI)-anchored protein, which binds folic acid and folate-linked molecules with high affinity and internalizes them via endocytosis. Imaging of FR-β can be realized with folate radiotracers. A number of folic acid-based radiotracers have been used pre-clinically for the imaging of activated macrophages in non-pulmonary, inflammatory conditions including e.g., rheumatoid arthritis, activated osteoarthritis, or atherosclerosis (41–47), FR-targeted radiopharmaceuticals have, however, not been evaluated yet in the context of ILD. Furthermore, the number of clinical studies making use of FR-targeting nuclear imaging strategies is limited including one exploratory trial in rheumatoid arthritis patients (48), since no folate-based radiotracer for PET imaging is currently available for clinical application.

A novel ^18^F-based folate PET radiotracer 3′-Aza-2′-[^18^F]-fluoro-folic acid, herein referred to as ^18^F-AzaFol), has recently been developed at the Center for Radiopharmaceutical Sciences ETH-PSI-USZ for FR imaging. The rationale to test this radiotracer instead of previously investigated macrophage imaging markers such as translocator protein (TSPO) was based on several disadvantages compared with ^18^F-AzaFol: TSPO (a) is mainly expressed in the outer mitochondrial membrane (49), thus it is not a cell surface receptor, (b) exhibits a high multicellular, basal expression in the lungs (50), and (c) TSPO-targeting PET tracers are still facing difficulties for clinical implementation. While the first generation of TSPO PET tracers showed high non-specific binding due to their lipophilic character (51), newer TSPO targeted radiotracers with improved binding specificity and affinity have still limitations due to the allelic dependency of the binding capability resulting from TSPO polymorphisms (52–54).

In this preclinical proof-of-concept study, we aimed to evaluate the potential of ^18^F-AzaFol-PET/CT for the specific visualization of macrophage-driven pathophysiologic processes in experimental ILD.

## Methods

### Human Subjects

Surgical lung biopsies from patients with IPF (*n* = 39) and CTD-ILD (*n* = 14), who underwent lung transplantation, were analyzed for the expression of FR-β. Lung sections from excess tissue from lung organ donors served as controls (*n* = 26). The patients' characteristics including demographic and clinical data are summarized in the data supplement (Supplementary Table 1).

The local ethics committee approved the study (BASEC-No. 2017-01298), and informed consent was obtained from all patients.

### Murine Model of Bleomycin-Induced Lung Fibrosis

As a representative animal model for experimental ILD, we used the well-established mouse model of BLM-induced lung fibrosis in this study. In the BLM model, inflammation peaks around day 7, whereas fibrosis reaches its maximum between days 14-21 (33, 35, 55). M1-like macrophages dominate the early inflammatory phase, whereas M2-like macrophages are most abundant in the pro-fibrotic phase, although they might appear as early as day 7 (56, 57).

Female C57BL/6J-rj mice (5–7 weeks old) were purchased from Janvier (Le Genest-Saint-Isle, France) and housed at the institutional animal facilities under defined temperature, humidity, and light conditions, and received *ad libitum* a standard rodent diet. After an acclimatization period of at least 7 days, lung fibrosis was induced in 8-week-old mice by instilling intratracheally a single dose of bleomycin sulfate (4 U/kg of body weight, Baxter, cantonal pharmacy Zurich, Switzerland) dissolved in sterile saline solution under isoflurane anesthesia (33–35). Control mice received equivalent volumes of 0.9% NaCl (50 μl). At days 3, 7, and 14 after the BLM instillation, biodistribution, and imaging studies were performed. Perfused lungs of separate animals were harvested for immunostainings, histological, and molecular analyses.

All animal experiments performed in this study were approved by the cantonal veterinary offices and conducted in strict compliance with the Swiss animal welfare guidelines. For all experiments, mice were randomized into the different study groups in a non-blinded manner.

### Histology

For histology, perfused middle, caudal, and accessory lobes of the right mouse lung were inflated with 10% neutral-buffered formalin solution and fixed overnight at room temperature (RT). After embedding in paraffin, lung sections were cut at a thickness of 4 μm and stained with hematoxylin and eosin (HE) for analysis of the lung architecture and the presence of cellular infiltrates, and with Picrosirius Red to detect collagen deposition using standard protocols.

### Immunohistochemistry on Murine Lung Tissues

For immunohistochemistry (IHC) on murine tissues, lung sections were deparaffinized and rehydrated, and then subjected to heat-mediated antigen retrieval with 10 mM sodium citrate buffer (pH = 6.0) at 95°C for 15 min. After blocking of endogenous peroxidase activity with 3% hydrogen peroxide (15 min, RT), sections were blocked with 10% normal goat serum (1 h, RT) followed by blocking of endogenous biotin using an Avidin/Biotin blocking kit (Vector Laboratories, Burlingame, CA, United States). Afterwards, primary antibodies for F4/80 (rat anti-mouse F4/80, clone Cl:A3-1, 1:100, AbD Serotec; Kidlington, United Kingdom), and FR-β (rabbit anti-mouse FR-β, 1:400, Genetex, Irvine, CA, United States) were applied on the specimens and incubated overnight at 4°C. Isotype- and concentration-matched IgGs served as negative controls. Next, biotin-labeled goat anti-rat or anti-rabbit secondary antibodies (all from Vector Laboratories) were applied (30 min, RT). This was followed by incubation with the Vectastain ABC Elite HRP kit for 30 min at RT (Vector Laboratories). Finally, stainings were visualized using 3,3′-diaminobenzidine (DAB) in case of F4/80, or 3-amino-9-ethylcarbazole (AEC) (all from Vector Laboratories) in case of FR-β, and sections were counterstained with Mayer's hematoxylin (J.T. Baker, Deventer, Netherlands).

### Immunohistochemistry on Human Lung Tissues

Immunohistochemistry was performed using an automated single-staining procedure (Benchmark Ultra; Ventana Medical Systems). Briefly, 4 μm thick sections were stained using mouse monoclonal anti-human antibodies directed against CD68 (clone PG-M1, Dako, 1:50) and FR-β (clone OTI8G1, Origen, 1:50). Detection was completed with respective secondary antibodies and the OptiView DAB Kit (Ventana Medical Systems).

### Immunofluorescence

Immunofluorescence (IF) stainings were performed using the MaxDouble IF staining kits for rat and rabbit primary antibodies (MaxVision Biosciences Inc., Bothell, WA, United States). In brief, after blocking of auto-fluorescence for 5 min at RT, heat-mediated antigen retrieval with 10 mM sodium citrate buffer was performed for 15 min at 95°C. After antigen retrieval, primary antibodies for FR-β (rabbit anti-mouse FR-β, 1:800, Genetex), and F4/80 (rat anti-mouse F4/80, clone Cl:A3-1, 1:800, AbD Serotec), or concentration matched IgG isotype controls were applied and incubated overnight at 4°C. Next, specimens were incubated with rat and rabbit signal amplifier for 30 min at RT followed by washing and linkage to the respective fluorophores for 60 min at RT in the dark (anti-rat MaxFluor488 and anti-rabbit MaxFlour594). Cell nuclei were counterstained with 4′,6-diamidino-2-phenylindole (DAPI).

### Microscopy and Image Analysis

Histological and immunohistochemical stainings were recorded automatically with the AxioScan.Z1. slidescanner (Carl Zeiss, Feldbach, Switzerland) using a Plan-Apochromat 20×/0.8 M27 objective. For semi-quantitative expression analyses, per sample, six randomly selected high power fields were extracted with a 10× objective using the *Zen 2.0 lite* (blue edition) software. The percentage of positively stained pixels was automatically quantified using an in-house designed *MATLAB* script (Mathworks, *MATLAB* R2016b) to avoid observer bias. This script quantified the target-positive (= brown or red) pixels and cell nuclei-positive (= blue) pixels and calculated the percentage of positively stained pixels in relation to the total number of image pixels. To account for increased cell numbers and tissue consolidations, also the percentage of positively stained pixels in relation to the total number of colored image (brown or red + blue) pixels was calculated (Supplementary Figure 1).

For semi-quantitative assessment of murine and human lung fibrosis, the Ashcroft Score was applied on Picrosirius Red stained lung sections as described previously (58). Two blinded examiners performed the scorings in duplicates. If deviations of more than 1 score were observed, the respective slides were re-assessed to reach consensus.

Immunofluorescent pictures were recorded at 630× magnification (oil immersion) using the Olympus BX53 microscope in fluorescence mode (Olympus, Volketswil, Switzerland).

The total number of double positive (FR-β+/F4/80+) cells was quantified by both automated and manual image analyses using Orbit image analyses software version 3.15 (Objection Detection and Object Classification Module) (59) or manual counting by two blinded examiners, respectively.

### RNA Extraction and Quantitative Reverse Transcription PCR

For RNA extraction from mouse lungs, perfused cranial lobes were homogenized using the Qiagen TissueLyser and total RNA was isolated with the RNeasy Fibrous Tissue Mini Kit from Qiagen (Hombrechtikon, Switzerland). For quantitative reverse transcription PCR (RT-qPCR), 120 ng RNA were reverse-transcribed into complementary DNA with the Transcriptor First Strand cDNA Synthesis Kit from Roche (Basel, Switzerland) using anchored-oligo(dT)_18_ primer. Messenger RNA (mRNA) expressions were analyzed by SYBR Green qPCR on a Stratagene Mx3005P qPCR System (Agilent Technologies, Santa Clara, California, USA) using the SYBR Green GoTaq qPCR Master mix from Promega (Dübendorf, Switzerland) and specific primers for murine *Folr2* (forward primer: 5′-CCAGCAAGTGGACCAGAGTT-3′, reverse primer: 5′-CAGTCCCAGCCTTTATGCCA-3′; Microsynth, Balgach, Switzerland). As a housekeeping gene 60S acidic ribosomal protein P0 (*Rplp0;* forward primer: 5′-GCAGGTGTTTGACAACGGCAG-3′, reverse primer: 5′-GATGATGGAGTGTGGCACCGA-3; Microsynth) was used. The fold change of mRNA expression was calculated using the 2^−ΔΔ*Ct*^ method. False positive results due to primer dimers or genomic contamination were excluded by dissociation curve analysis and non-template controls, or by minus-reverse transcriptase controls, respectively.

### Hydroxyproline Assay

Collagen contents in lungs of BLM-treated mice and saline controls were quantified by hydroxyproline assay as described previously (60). Briefly, after homogenization, left lung lobes were digested in 6 M HCl for 3 h at 120°C and subsequently neutralized with 6 M NaOH. Next, samples were mixed with a 60 mM chloramine T solution and incubated for 20 min at RT. After addition of 3.15 M perchloric acid (5 min, RT), p-Dimethylaminobenzaldehyd (20% w/v) was added and samples were incubated for 20 min at 60°C. The absorbance was measured at 560 nm with a spectrophotometer (*GloMax-Multi Detection System*, Promega, Dübendorf, Switzerland).

### Radiosynthesis of ^18^F-AzaFol

3′-Aza-2′-[^18^F]-fluoro-folic acid (^18^F-AzaFol) was produced on an automated synthesis module at the ETH Zurich (Switzerland) according to a previously reported method (61). ^18^F-AzaFol was applied at ~5 MBq (in 100 μL, 0.25–0.5 pmol/mouse) for biodistribution studies and at ~10 MBq (in 100 μL, 0.5–1 pmol/mouse) for *in vivo* and *ex vivo* PET/CT imaging. The *in vivo* stability of the tracer has been demonstrated in previous studies, in which only the intact parent radiotracer was detected. No downstream radiometabolites in blood plasma, urine, or liver samples were detectable (61, 62).

### Biodistribution Studies

As an accurate means to quantify radiotracer uptake (31), biodistribution studies were performed. ^18^F-AzaFol was administrated via the lateral tail vein of mice. Receptor-blocking studies were performed by pre-injection of folinic acid (leucovorin; 300 nmol, 100 μL) ~30 min before the injection of ^18^F-AzaFol. Mice were sacrificed 1 h post injection (p.i.) of ^18^F-Azafol. Tissues and organs of interest were collected, weighed and counted for activity using a γ-counter (*Wallac Wizard 1480*, Perkin Elmer, Germany). The results were calculated as a percentage of the injected activity per gram of tissue mass (% IA/g) or expressed as a percentage of the injected activity per organ (% IA/organ). Thereafter, the already counted lungs were subjected to *ex vivo* PET/CT scans as described below.

### *Ex vivo* and *in vivo* PET/CT Scans

The *ex vivo* PET/CT scans of collected lungs (obtained from mice used for the biodistribution study) were performed using a small-animal bench-top PET/CT scanner (*G8*, Perkin Elmer, Massachusetts, USA; Table 1). Static PET scans of 20 min duration were acquired using the G8 acquisiton software (version 2.0.0.10) followed by CT scans of 1.5 min duration. The energy window ranged from 150 to 650 keV. The PET data were corrected for random coincidences, decay, and dead time and reconstructed with maximum-likelihood expectation maximization (MLEM). A correction for scatter was not made. The images were prepared using VivoQuant post-processing software (version 3.0, inviCRO Imaging Services and Software, Boston, USA). A Gauss post-reconstruction filter (FWHM = 1 mm) was applied to the PET images. The *ex vivo* PET/CT scans and biodistribution studies were performed in a separate experiment as the *in vivo* PET/CT scans. For *in vivo* imaging, static PET scans were performed 1 h p.i. of the radiotracer and lasted for 10 min followed by a CT of 1.5 min duration. During the *in vivo* PET/CT scans, the mice were anesthetized with a mixture of isoflurane and oxygen. The *in vivo* images were visualized using a dedicated 3D-rendering software (Ziostation2, Ziosoft, Tokyo, Japan).

**Table 1:** Scan parameters for *in vivo* and *ex vivo* PET scans using *G8* bench-top PET/CT scanner.

Energy window	150–650 keV
Isotope	Fluorine 18
Framing sequence	Static
Duration of static PET scans	
*In vivo* (chest region)	10 min
*Ex vivo* (isolated whole lungs)	20 min
Normalization	Yes
Dead time correction	Yes
Decay correction	Yes
Scatter correction	No
Image reconstruction	
Number of iterations	60
Attenuation correction	Yes

*In vivo and ex vivo scans were performed 1 h after the injection of ^18^F-AzaFol (~10 MBq in 100 μL, 0.5–1 pmol/per mouse)*.

For both, biodistribution studies/*ex vivo* scans and the *in vivo* scans per each time point the following numbers of mice have been used: *n* = 3–4 for saline-treated mice, *n* = 3–4 for BLM-treated mice and *n* = 2–4 for BLM-treated mice receiving leucovorin for FR blockade.

### Statistics

Statistical analysis was performed using GraphPad Prism 7 software (version 7.04). Unless otherwise indicated, non-parametric data were expressed as median ± interquartile range (IQR) and parametric data were expressed as mean ± standard deviation (S.D.). For non-parametric non-related data, the Mann–Whitney *U*-test for comparison of two groups, or the Kruskal–Wallis test followed by Dunn's multiple correction for comparison of multiple groups was employed. For parametric non-related data, an unpaired *t*-test was applied for comparison between two groups, or a One-Way ANOVA with Tukey's *post-hoc* test for comparison between multiple groups was performed. For correlation analysis, Spearman rank correlation was performed. *P*-values < 0.05 were considered statistically significant.

## Results

### FR-β Expression Is Upregulated in Human ILD and Correlates With Disease Severity

To assess the presence of FR-β-positive macrophages in human ILD, we performed IHC for FR-β on lung explants derived from patients with IPF (*n* = 39) and CTD-ILD (*n* = 14), who underwent lung transplantation. Lung sections from excess tissue from lung organ donors served as controls (*n* = 26) (Supplementary Table 1).

**Figure 1:**
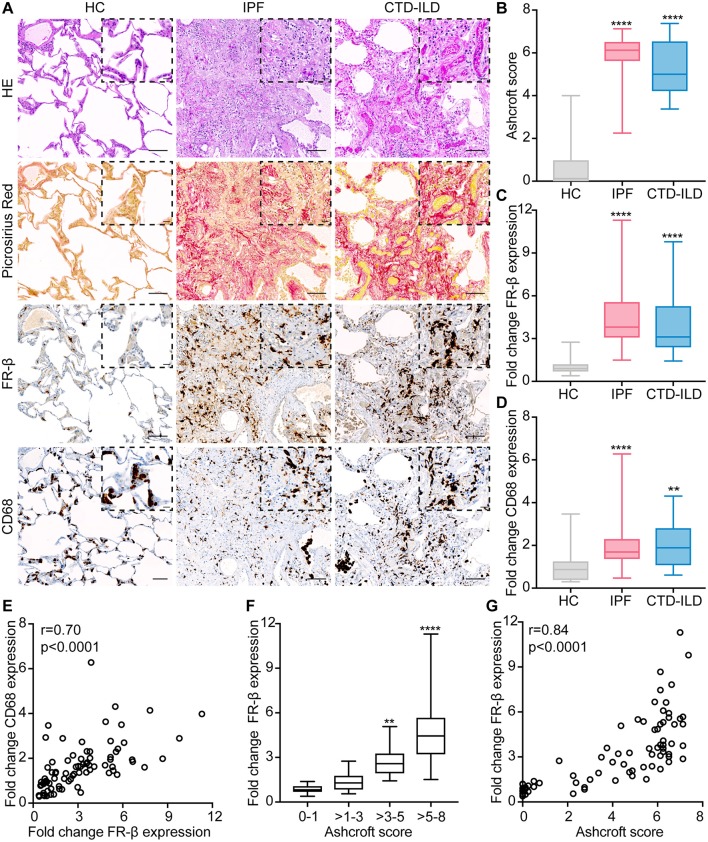
FR-β expression is increased in human ILD and correlates with the severity of lung remodeling. **(A)** Representative images of lung sections from healthy controls (HC) and patients with IPF and CTD-ILD stained with hematoxylin and eosin (HE, first panel), Picrosirius Red (collagen = red, second panel), FR-β (brown, third panel) and CD68 (brown, fourth panel). Representative pictures at 100× magnification (scale bars: 100 μm) and at higher magnification (400×, scale bars: 20 μm) are shown. **(B)** Semi-quantification of pulmonary fibrosis by Ashcroft score. **(C)** Semi-quantification of FR-β tissue expression by automatic image analysis. **(D)** Semi-quantification of CD68 tissue expression by automatic image analysis. **(E)** Spearman correlation of FR-β expression with the CD68 expression. **(F)** Analysis of FR-β expression according to the severity of lung remodeling as defined by the Ashcroft score (score 0–1: no fibrosis, scores >1–3: mild fibrosis, scores >3–5: moderate fibrosis, scores >5–8: severe fibrosis). **(G)** Spearman correlation of FR-β expression with the Ashcroft score. For **(B–D,F)** data are displayed as box plots with min/max values. For statistical analysis, the Kruskal–Wallis test with Dunn's multiple correction was applied (*p < 0.05, **p < 0.01, ***p < 0.001, ****p < 0.0001). For all experiments: *n* = 26 for healthy controls, *n* = 39 for IPF patients, and *n* = 14 for CTD-ILD patients.

**Figure 2:**
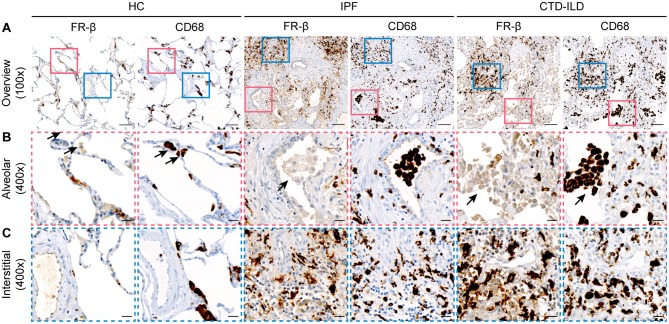
FR-β is mostly expressed in the lung interstitium in human ILD. Representative images of sequential lung sections from healthy controls (HC, *n* = 26) and patients with IPF (*n* = 39) and CTD-ILD (*n* = 14) that were stained with FR-β and the human macrophage marker CD68. **(A)** Overview images at 100× magnification are shown (scale bars: 100 μm). Colored squares indicate regions of interest for the higher magnification images depicted in **(B,C)**. **(B)** Higher magnification images at 400× magnification (scale bars: 20 μm) indicating the only weak to undetectable expression of FR-β on macrophages located in alveolar spaces (arrows). **(C)** Higher magnification images at 400× magnification (scale bars: 20 μm) indicating the strong expression of FR-β on macrophages located in the lung interstitium.

As anticipated, the histopathological analysis of lung tissues from both patients with IPF and CTD-ILD revealed severe damage of the normal tissue architecture with increased numbers of mononuclear inflammatory infiltrates and excessive interstitial collagen deposition as assessed by HE or Picrosirius Red staining (Figure 1A), respectively. This was also reflected in the semi-quantitative Ashcroft score of pulmonary fibrosis with a median score of 6.125 (Q1, Q3 = 5.625, 6.5; *p* < 0.0001) for IPF and a median score of 5 (Q1, Q3 = 4.219, 6.531; *p* < 0.0001) for CTD-ILD patients (Figure 1B). In most cases, lung remodeling in IPF and CTD-ILD patients had histological features of usual interstitial pneumonia (UIP) characterized by patchy fibrosis and areas of honeycombing, whereas only the minority of patients displayed patterns of non-specific interstitial pneumonia (NSIP), characterized by a more uniformly spread fibrosis and better preserved lung architecture (Supplementary Table 1). In these highly inflammatory and fibrotic lung sections, presence of FR-β was significantly increased (Figure 1A) with median increases of ~ 3- to 4-fold in both IPF and CTD-ILD patients (Figure 1C, Supplementary Figure 2A; *p* < 0.0001). To confirm the expression of FR-β on macrophages (40, 63), we additionally performed IHC for CD68, a human pan-macrophage marker, on sequential lung sections from healthy controls, IPF and CTD-ILD patients. In accordance with the increased FR-β expression, we also found increased CD68 expression in lung sections from both IPF and CTD-ILD patients with median increases of 1.69-fold (Q1, Q3 = 1.37, 2.29; *p* < 0.0001) and 1.89-fold (Q1, Q3 = 1.09, 2.8; *p* < 0.01), respectively (Figure 1D, Supplementary Figure 2B). Consistently, FR-β expression also strongly correlated with the CD68 expression (*r* = 0.70, *p* < 0.0001; Figure 1E). Whereas, a strong expression of CD68 was observed on macrophages in the alveolar spaces, FR-β was mostly expressed in the lung interstitium (Figure 2). Furthermore, while the upregulation of CD68 and FR-β in IPF and CTD-ILD patients was independent of the histological subtype (Supplementary Figure 3), the expression of FR-β significantly increased with the severity of lung remodeling (Figure 1F) and positively correlated with the Ashcroft score as measure of lung remodeling (*r* = 0.84, *p* < 0.0001; Figure 1G).

### FR-β Expression Is Also Upregulated in Experimental ILD and Changes With Disease Development

Next, we assessed whether the expression of FR-β was mirrored in a representative mouse model of human ILD, the BLM-induced lung fibrosis model.

**Figure 3:**
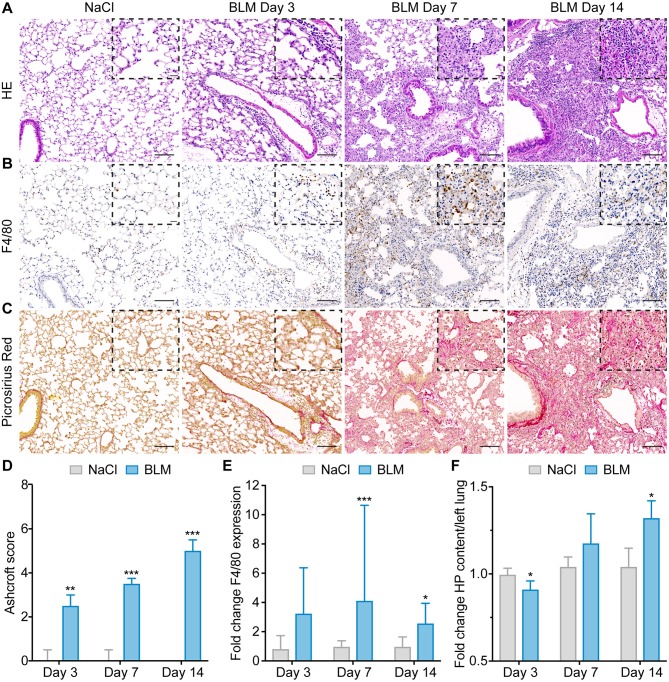
Intratracheal instillation of bleomycin in mice induces progressive lung remodeling characterized by increased numbers of macrophages. **(A)** Representative images of lung sections from saline-treated controls and BLM-treated mice at days 3, 7, and 14 stained with hematoxylin and eosin (HE), **(B)** the murine macrophage marker F4/80 (brown), and **(C)** Picrosirius Red (collagen = red). **(D)** Semi-quantitative assessment of lung fibrosis by Ashcroft score. **(E)** Semi-quantification of pulmonary macrophages by automatic image analysis of F4/80 expression. **(F)** Quantification of left lung collagen content by hydroxyproline (HP) assay. For **(A–C)** representative pictures at 100× magnification (scale bars: 100 μm) and at higher magnification (400×, scale bars: 20 μm) are shown. For **(D–F)** data are presented as medians ± IQR. For statistical analysis, the Mann–Whitney U-test was applied (*p < 0.05, **p < 0.01, ***p < 0.001). For all experiments: *n* = 4–6 for saline controls and *n* = 6–10 for BLM-treated mice.

Upon a single intratracheal BLM administration, mice progressively developed lung remodeling with inflammation (days 3–7) preceding the development of pulmonary fibrosis (day 14). As early as day 3, the histopathological examination of lung sections from BLM-treated mice vs. saline controls revealed the presence of mononuclear cell infiltrates (Figure 3A) around the vessels and bronchi with increased numbers of macrophages as assessed by IHC with the murine macrophage marker F4/80 (Figure 3B). In contrast, only minimal fibrous thickening of the alveolar and bronchial walls (Figure 3C) was detected, which was also reflected by a low median Ashcroft score of 2.5 (Q1, Q3 = 2, 3; *p* < 0.01; Figure 3D). With disease progression, the number of macrophages increased in BLM-treated lungs and peaked at day 7 with a median 4.11-fold increase (Q1, Q3 = 2.38, 10.65; *p* < 0.001; Figure 3E, Supplementary Figure 2D) and subsided thereafter. Consistently, pulmonary fibrosis gradually increased with extensive interstitial collagen deposition characterized by the formation of fibrous bands and larger fibrous masses in subpleural as well as perivascular and peribronchial areas (Figure 3C). Maximally established fibrosis was detected at day 14 as demonstrated by a high median Ashcroft score of 5 (Q1, Q3 = 4.25, 5.5; *p* < 0.001). The lung collagen content as assessed by hydroxyproline (HP) assay increased over time in BLM-treated mice with a median 1.32-fold (Q1, Q3 = 1.17, 1.42; *p* < 0.05) increase at day 14 (Figure 3F).

**Figure 4:**
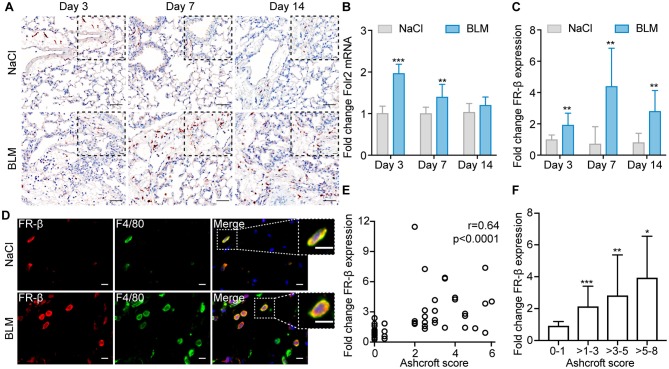
FR-β expression is increased in experimental ILD and correlates with the severity of lung remodeling. **(A)** Representative images of lung sections from saline-treated controls and BLM-treated mice at days 3, 7, and 14 stained for FR-β (red). Representative pictures at 100× magnification (scale bars: 100 μm) and at higher magnification (400×, scale bars: 20 μm) are shown. **(B)** Fold change of mRNA expression of *Folr2* in BLM-treated mice vs. saline-treated controls at days 3, 7, and 14. **(C)** Semi-quantification of FR-β tissue expression by automatic image analysis. **(D)** Representative images of immunofluorescent double staining of FR-β (red) with the murine macrophage marker F4/80 (green) performed on lung sections from saline-treated controls and BLM-treated mice at day 7. For **(D)** representative images from three mice each at 630× magnification are shown (scale bars: 10 μm). **(E)** Spearman correlation of FR-β expression with the Ashcroft score. **(F)** Analysis of FR-β expression according to the severity of lung remodeling as defined by the Ashcroft score (score 0–1: no fibrosis, scores >1–3: mild fibrosis, scores >3–5: moderate fibrosis, scores >5–8: severe fibrosis). For **(B,C,F)** data are presented as medians ± IQR. For statistical analysis the Kruskal–Wallis test with Dunn's multiple correction or the Mann–Whitney *U*-test was applied (*p < 0.05, **p < 0.01, ***p < 0.001). For all experiments: *n* = 6 for saline-treated controls, *n* = 9–10 for BLM-treated mice.

In accordance with the expression in human ILD and the time course of the appearance of pulmonary macrophages in this animal model, FR-β expression significantly increased over time in lungs of BLM-treated mice (Figure 4A). While FR-β was only weakly expressed in the lungs of saline-treated controls, FR-β expression was significantly upregulated in lungs of BLM-treated mice with peak at day 3 at the mRNA level (Figure 4B) and at day 7 at the protein level (Figures 4A,C, Supplementary Figure 2C) and thus in the inflammatory phase in this animal model with a median increase of 1.98-fold (Q1, Q3 = 1.61, 2.19; *p* < 0.001) and 4.41-fold (Q1, Q3 = 2.4, 6.83; *p* < 0.01), respectively. The expression of FR-β on murine lung macrophages was confirmed by immunofluorescent double staining using an antibody for murine macrophages, F4/80 (Figure 4D, Supplementary Figure 4).

As in human ILD patients, the expression of FR-β also positively correlated with the Ashcroft score (*r* = 0.64, *p* < 0.0001; Figure 4E) and, hence, with the degree of lung remodeling (Figure 4F).

### Pulmonary Accumulation of ^18^F-AzaFol, a Surrogate Marker for FR-β-Positive Macrophages, Reflects Macrophage-Related Disease Development in Experimental ILD

Having established the time course of FR-β expression in this mouse model, we next performed nuclear imaging experiments to assess whether macrophage-related disease development could be visualized by ^18^F-AzaFol.

**Figure 5:**
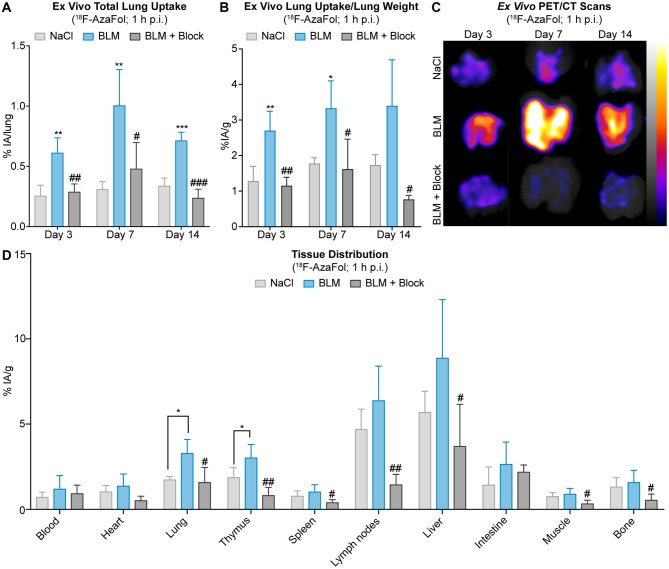
Pulmonary accumulation of ^18^F-AzaFol reflects macrophage-related disease development in experimental ILD. **(A)** *Ex vivo* lung uptake of ^18^F-AzaFol (1 h p.i.) shown as percentage of injected activity per lung (% IA/lung) or **(B)** shown as percentage of injected activity per lung mass (% IA/g) in lungs from saline-treated controls and BLM-treated mice with and without receptor blockade at days 3, 7, and 14. **(C)** *Ex vivo* PET/CT scans of lungs from saline controls and BLM-treated mice with and without receptor blockade at days 3, 7, and 14 that were collected 1 h after injection of ^18^F-AzaFol. Scans are shown as maximum intensity projections. **(D)** Representative tissue distribution of ^18^F-AzaFol (1 h p.i.) in organs of interest of saline-treated controls and BLM-treated mice. Biodistribution data are shown for day 7, when the strongest pulmonary accumulation of ^18^F-AzaFol was observed. Data are expressed as percentage of injected activity per gram of tissue (% IA/g). For **(A,B,D)** data are presented as means ± S.D. For statistical analysis, the One Way ANOVA test with Tukey's multiple correction was applied (*p < 0.05, **p < 0.01, ***p < 0.001, vs. saline; ^#^*p* < 0.05, ^*##*^*p* < 0.01, ^*###*^*p* < 0.001, vs. BLM). For all experiments: *n* = 3–4 for saline-treated controls, *n* = 3–4 for BLM-treated mice, and *n* = 2–4 for BLM-treated mice receiving receptor blockade.

In strong correlation with the expression changes of FR-β at the tissue level, BLM-treated mice showed increased pulmonary accumulation of ^18^F-AzaFol from days 3 to 14, as assessed by *ex vivo* biodistribution studies at 1 h p.i. of ^18^F-AzaFol (Figures 5A,B). The maximum lung accumulation was observed at day 7 with a mean total uptake of 1.01 ± 0.30% injected activity per lung (% IA/lung) and 3.33 ± 0.77% injected activity per tissue mass (% IA/g) in BLM-treated vs. control animals (0.31 ± 0.06% IA/lung and 1.78 ± 0.15% IA/g; *p* < 0.01 and *p* < 0.05, respectively). The specificity of the pulmonary tissue uptake of ^18^F-AzaFol was validated by receptor blockade using folinic acid (leucovorin) administrated to mice 30 min prior to the injection of the radiotracer. This significantly reduced the lung accumulation of ^18^F-AzaFol in BLM-treated mice, resulting in pulmonary radioactivity accumulation comparable to saline-treated controls. *Ex vivo* PET/CT scans of isolated lungs also clearly distinguished diseased from healthy lungs and confirmed the successful receptor blockade (Figure 5C). *In vivo* PET/CT scans of the chest region of the animal showed a generally low pulmonary accumulation of ^18^F-AzaFol with background signals in bone and muscles in both NaCl-treated controls and BLM-treated mice. A slightly increased signal intensity compared with control mice was observed in lungs of BLM-treated mice at the maximum of inflammation at day 7 (Supplementary Figure 5).

The distribution of ^18^F-AzaFol in other organs besides the lungs was comparable between BLM-treated mice and saline-treated control animals at day 7 (Figure 5D, Supplementary Tables 2, 3). The slightly increased uptake in both experimental groups in lymphoid organs (e.g., thymus, lymph nodes), which may have been also affected by the BLM treatment (64), is most likely caused by the presence of activated macrophages expressing FR–β (65, 66), a phenomenon, previously observed in lymph nodes of tumor-bearing mice (61). The high basal liver uptake of ^18^F-AzaFol observed in both controls and BLM-treated mice can be explained by the fact that folate vitamins are physiologically stored in the liver. Since ^18^F-AzaFol is not a conjugate of folic acid, as this is the case with other folate radioligands, ^18^F-AzaFol may also be transported through carrier systems such as the proton-coupled folate transporter. A relatively high liver uptake and potential signs of metabolism were also shown in our previous studies, in which we evaluated ^18^F-AzaFol for the first time (61).

The distinct drop in activity after treatment with the FR blocking agent in both liver and lymph nodes further argues for a folate-specific effect rather than an unspecific accumulation mechanism.

## Discussion

In different types of ILD, an increased lung uptake of [^18^F]FDG was observed in pathologically changed areas of reticulation/honeycombing and ground-glass opacities, but also in radiologically normal-appearing lung areas (28, 29, 67–71). [^18^F]FDG uptake, especially in normal appearing lung parenchyma, was shown to be of prognostic value and to correlate with overall disease severity in IPF patients (28, 68, 70). However, [^18^F]FDG-PET/CT has also important limitations for the diagnosis and monitoring of ILD since it visualizes changes in glucose metabolism in a non-specific, cell-type-independent manner (25, 26, 67), and does not allow to draw conclusions on the pathophysiological disease stage (30, 72). In our study, ^18^F-AzaFol visualized macrophage-related ILD development in the mouse model of BLM-induced lung fibrosis in *ex vivo* PET/CT scans and tissue expression of FR-β showed good correlation with the pulmonary radiotracer uptake in the biodistribution studies. This has important clinical implications.

As ILDs are highly heterogeneous on molecular level, such a targeted molecular imaging approach could be used in the future for a molecular stratification of ILD patients, i.e., the identification of subgroups of patients, who are likely to benefit from macrophage-oriented therapies and who will be eligible for subsequent monitoring of therapeutic responses. This is of particular interest due to the recent or imminent approval of such therapies for ILD (73–75). These include pirfenidone and nintedanib, which have pre-clinically shown to exert their anti-fibrotic effects at least partially by targeting pulmonary macrophages and/or their products (75–77) as well as tocilizumab, which was assessed in recent phase II and III randomized controlled studies (78). In addition, the ability to identify ILD patients based on their underlying molecular and cellular subtype without the need of lung biopsies might also have relevance for clinical trial design by allowing the definition of (more) homogenous patients' subgroups and by serving as a primary/secondary readout for macrophage-orientated treatment studies. To this end, additional preclinical studies to confirm the suitability for ^18^F-AzaFol for predicting and monitoring therapeutic efficacy will have to be performed. The alterations of tissue uptake of ^18^F-AzaFol throughout the development of experimental ILD were in strong accordance with the time course of macrophage presence on tissue level, which documents a good sensitivity to change. This quality is an important prerequisite for the monitoring of macrophage-targeted treatment responses, which we have not yet tested pre-clinically.

Another important finding of this study was that the numbers of (FR-β-positive) macrophages were substantially increased in human ILD patients, irrespective of the underlying etiology. This observation has some interesting implications. Firstly, it supports the re-evaluation of the pathophysiology of fibrotic ILD as immune-mediated and thus, as potentially amenable to immune-targeted therapies (12). Secondly, the persistence of macrophages throughout different stages of experimental ILD and their presence in late disease stages in the human disease points to an important role in the whole process of tissue remodeling (79). Thus, the characterization of macrophage subpopulations, particularly of FR-β-positive macrophages, might further elucidate the mechanisms of fibrosis in ILD and identify novel macrophage- or macrophage-related therapeutic targets (15) including FR-β-targeted molecular therapies (40, 80–82).

The exploratory character of our study accounts for some of its limitations. The model of BLM-induced lung fibrosis, although extensively used and widely acknowledged as a valuable model of experimental ILD, does not reflect the chronic disease course in human patients, since following the single instillation of BLM, fibrosis gradually resolves over 4–8 weeks (55). Furthermore, the imaging analyses have been limited to the stages of active disease and later time points of resolution of fibrosis (days 21–28) have not been investigated. For pathophysiologic studies to elucidate the whole process of macrophage-related tissue remodeling in ILD in detail, FR-β-targeted nuclear imaging would have to be performed (a) in the phase of tissue repair in the acute BLM model and (b) in non-resolving, chronic disease models of ILD (83). Numerous studies, however, are now focusing on the acute, pro-inflammatory phase and the role of macrophages in fibrosis development in this model (15, 56, 57), which support the importance and comparability of its early stages to certain aspects of human ILD.

Another important limitation of our proof-of-concept study is that our nuclear imaging experiments are largely based on *ex vivo* analyses and quantifications. Furthermore, the performance of static PET scans did not allow the correction for changes in pulmonary blood flow. Elevated blood flow and increased vascular leakage are cardinal features of lung inflammation and fibrosis (34, 84). These phenomena could have contributed non-specifically to the pulmonary accumulation of ^18^F-AzaFol in BLM-treated mice. The fact that receptor blockade with folinic acid lowered the radiotracer uptake to the level of control mice, however, points to a receptor-specific rather than a non-specific pulmonary uptake of ^18^F-AzaFol.

In future preclinical experiments to further support the specificity of ^18^F-AzaFol-PET/CT, (a) the quality of the *in vivo* imaging should be improved by using gated respiration during the acquisition of the nuclear images to reduce motion artifacts, which could affect tissue density, (b) dynamic PET scans should be performed to account for blood flow-related changes, and (c) signal intensities should be also quantified *in vivo* e.g., by calculating the standardized uptake values (84, 85). These additional studies would allow to better estimate the clinical applicability of ^18^F-AzaFol-PET/CT. For the extrapolation of our preclinical data to humans, it is further important to note that the endogenous folate levels largely differ between rodents and humans (~15–40 fold higher in mice). In our study, this could have led to an underestimation of the actual ^18^F-AzaFol tissue uptake since endogenous folate might also compete with ^18^F-AzaFol for binding to the FR (86).

In general, the transferability of results from animal models, which, even though representative of certain aspects, never cover the whole complexity of a human disease, is always a matter of debate. However, previous studies using the murine BLM-induced lung fibrosis model provided evidence for its suitability for both imaging (33, 87) and molecular analyses (88) and our own data showed similarly high pulmonary expression levels of FR-β in experimental and (end-stage) human ILD.

In conclusion, our proof-of-concept study showed that nuclear imaging using ^18^F-AzaFol can visualize macrophage-related experimental ILD. The fact that FR-β—apart from being a cellular rather than a metabolic marker—is only expressed on activated macrophages in disease states such as inflammatory disorders or malignancies, supports ^18^F-AzaFol as a more specific alternative to [^18^F]FDG in ILD. Since ^18^F-AzaFol-PET/CT has been tested for targeting FR-positive tumors in a Swiss multi-center trial (NCT03242993; www.clinicaltrials.gov), its clinical availability, including first-in-human clinical trials for imaging of ILD, is impending.

## Data Availability Statement

All datasets generated for this study are included in the article/Supplementary Material.

## Ethics Statement

The studies involving human participants were reviewed and approved by Ethics committee Zurich. The patients/participants provided their written informed consent to participate in this study. The animal study was reviewed and approved by Swiss veterinary office. The local ethics committee approved the study (BASECNo. 2017-01298), and informed consent was obtained from all patients.

## Author Contributions

JS made substantial contributions to the conception of the study and the acquisition, analysis and the interpretation of data, and was involved in drafting and revising the manuscript. MBe, MBr, SH, SC, TF, BV, and CF-B were centrally involved in the acquisition and analysis of data and in revising the manuscript. RS and OD made contributions to the conception and design of the study, the interpretation of the data, and the revision of the manuscript. CM and BM made substantial contributions to conception, design of the study and were centrally involved in the acquisition, analysis and interpretation of data, and in drafting and revising the manuscript.

### Conflict of Interest

^18^F-AzaFol is patent pending (WO 2013/167653 A1) and the patent is owned by Merck & Cie, Switzerland, an affiliate of Merck KGaA, Darmstadt, Germany. RS and CM are co-inventor on this patent. RS received funding for the development of ^18^F-AzaFol from Innosuisse (grant no.: CTI-Project 13877.1 PFLS-LS). CM received funding from Merck & Cie for the performance of preclinical studies with ^18^F-AzaFol. OD had consultancy relationships with Actelion, AnaMar, Bayer, Boehringer Ingelheim, Catenion, CSL Behring, ChemomAb, Roche, GSK, Inventiva, Italfarmaco, Lilly, medac, Medscape, Mitsubishi Tanabe Pharma, MSD, Novartis, Pfizer, Sanofi, and UCB in the area of potential treatments of scleroderma and its complications. Additionally, OD has research funding from Actelion, Bayer, Boehringer Ingelheim, Mitsubishi Tanabe Pharma, and Roche. In addition, OD has a patent mir-29 for the treatment of systemic sclerosis registered. BM had grant/research support from AbbVie, Protagen, Novartis, congress support from Pfizer, Roche, and Actelion. In addition, BM has a patent mir-29 for the treatment of systemic sclerosis registered. The real or perceived potential conflicts listed above are accurately stated. The remaining authors declare that the research was conducted in the absence of any commercial or financial relationships that could be construed as a potential conflict of interest.

## Abbreviations

ILD: Interstitial lung disease
PET/CT: Positron emission computed tomography/computed tomography
BLM: Bleomycin
IPF: Idiopathic pulmonary fibrosis
CTD-ILD: Connective tissue disease-associated interstitial lung disease
SSc: Systemic sclerosis
HRCT: High resolution computed tomography
MRI: Magnetic resonance imaging
[^18^F]FDG: [^18^F]fluorodeoxyglucose
FR-β: Folate receptor-β
GPI: Glycosylphosphatidylinositol
^18^F-AzaFol: 3′-Aza-2′-[^18^F]-fluoro-folic acid
RT: Room temperature
HE: Hematoxylin and eosin
IHC: Immunohistochemistry
DAB: Diaminobenzidine
AEC: 3-Amino-9-ethylcarbazole
IF: Immunofluorescence
DAPI, 4′: 6-Diamidino-2-phenylindole
RT-qPCR: Quantitative reverse transcription polymerase chain reaction
mRNA: Messenger RNA
Rplp0: 60S acidic ribosomal protein P0
p.i.: Post injection
% IA/g: Percentage of the injected activity per gram of tissue mass
% IA/organ: Percentage of the injected activity per organ
MLEM, Maximum-likelihood expectation maximization IQR: Interquartile range
S.D.: Standard deviation
UIP: Usual interstitial pneumonia
NSIP: Non-specific interstitial pneumonia
HP: Hydroxyproline.

## References

[1] Wynn TA. Fibrotic disease and the T(H)1/T(H)2 paradigm. Nat Rev Immunol. (2004) 4:583–94. 10.1038/nri1412 15286725 PMC2702150

[2] Hutchinson JP McKeever TM Fogarty AW Navaratnam V Hubbard RB. Increasing global mortality from idiopathic pulmonary fibrosis in the twenty-first century. Ann Am Thorac Soc. (2014) 11:1176–85. 10.1513/AnnalsATS.201404-145OC 25165873

[3] Hoffmann-Vold AM Fretheim H Halse AK Seip M Bitter H Wallenius M. Tracking impact of interstitial lung disease in systemic sclerosis in a complete nationwide cohort. Am J Respir Crit Care Med. (2019). 10.1164/rccm.201903-0486OC 31310156

[4] Long K Danoff SK. Interstitial lung disease in polymyositis and dermatomyositis. Clin Chest Med. (2019) 40:561–72. 10.1016/j.ccm.2019.05.004 31376891

[5] Brito Y Glassberg MK Ascherman DP. Rheumatoid arthritis-associated interstitial lung disease: current concepts. Curr Rheumatol Rep. (2017) 19:79. 10.1007/s11926-017-0701-5 29119259

[6] Tselios K Urowitz MB. Cardiovascular and pulmonary manifestations of systemic lupus erythematosus. Curr Rheumatol Rev. (2017) 13:206–18. 10.2174/1573397113666170704102444 28675998

[7] Roca F Dominique S Schmidt J Smail A Duhaut P Levesque H. Interstitial lung disease in primary Sjogren's syndrome. Autoimmun Rev. (2017) 16:48–54. 10.1016/j.autrev.2016.09.017 27682894

[8] Reiseter S Gunnarsson R Mogens Aalokken T Lund MB Mynarek G Corander J. Progression and mortality of interstitial lung disease in mixed connective tissue disease: a long-term observational nationwide cohort study. Rheumatology. (2018) 57:255–62. 10.1093/rheumatology/kex077 28379478

[9] Fischer A Brown KK. Interstitial lung disease in undifferentiated forms of connective tissue disease. Arthritis Care Res. (2015) 67:4–11. 10.1002/acr.22394 25048539

[10] Wallace B Vummidi D Khanna D. Management of connective tissue diseases associated interstitial lung disease: a review of the published literature. Curr Opin Rheumatol. (2016) 28:236–45. 10.1097/BOR.0000000000000270 27027811 PMC4826478

[11] Maher TM Wells AU Laurent GJ. Idiopathic pulmonary fibrosis: multiple causes and multiple mechanisms? Eur Respir J. (2007) 30:835–9. 10.1183/09031936.00069307 17978154

[12] Wells AU Denton CP. Interstitial lung disease in connective tissue disease–mechanisms and management. Nat Rev Rheumatol. (2014) 10:728–39. 10.1038/nrrheum.2014.149 25266451

[13] Sisson TH Mendez M Choi K Subbotina N Courey A Cunningham A. Targeted injury of type II alveolar epithelial cells induces pulmonary fibrosis. Am J Respir Crit Care Med. (2010) 181:254–63. 10.1164/rccm.200810-1615OC 19850947 PMC2817814

[14] Plataki M Koutsopoulos AV Darivianaki K Delides G Siafakas NM Bouros D. Expression of apoptotic and antiapoptotic markers in epithelial cells in idiopathic pulmonary fibrosis. Chest. (2005) 127:266–74. 10.1378/chest.127.1.266 15653994

[15] Byrne AJ Maher TM Lloyd CM. Pulmonary macrophages: a new therapeutic pathway in fibrosing lung disease? Trends Mol Med. (2016) 22:303–16. 10.1016/j.molmed.2016.02.004 26979628

[16] Aran D Looney AP Liu L Wu E Fong V Hsu A. Reference-based analysis of lung single-cell sequencing reveals a transitional profibrotic macrophage. Nature Immunol. (2019) 20:163–72. 10.1038/s41590-018-0276-y 30643263 PMC6340744

[17] Christmann RB Sampaio-Barros P Stifano G Borges CL de Carvalho CR Kairalla R. Association of interferon- and transforming growth factor beta-regulated genes and macrophage activation with systemic sclerosis-related progressive lung fibrosis. Arthritis Rheumatol. (2014) 66:714–25. 10.1002/art.38288 24574232 PMC4439004

[18] Schupp JC Binder H Jager B Cillis G Zissel G Muller-Quernheim J. Macrophage activation in acute exacerbation of idiopathic pulmonary fibrosis. PLoS ONE. (2015) 10:e0116775. 10.1371/journal.pone.0116775 25590613 PMC4295887

[19] Hsu E Shi H Jordan RM Lyons-Weiler J Pilewski JM Feghali-Bostwick CA. Lung tissues in systemic sclerosis have gene expression patterns unique to pulmonary fibrosis and pulmonary hypertension. Arthritis Rheum. (2011) 63:783–94. 10.1002/art.30159 21360508 PMC3139818

[20] Cai M Bonella F He X Sixt SU Sarria R Guzman J. CCL18 in serum, BAL fluid and alveolar macrophage culture supernatant in interstitial lung diseases. Respir Med. (2013) 107:1444–52. 10.1016/j.rmed.2013.06.004 23831213

[21] Mahoney JM Taroni J Martyanov V Wood TA Greene CS Pioli PA. Systems level analysis of systemic sclerosis shows a network of immune and profibrotic pathways connected with genetic polymorphisms. PLoS Comput Biol. (2015) 11:e1004005. 10.1371/journal.pcbi.1004005 25569146 PMC4288710

[22] Taroni JN Greene CS Martyanov V Wood TA Christmann RB Farber HW. A novel multi-network approach reveals tissue-specific cellular modulators of fibrosis in systemic sclerosis. Genome Med. (2017) 9:27. 10.1186/s13073-017-0417-1 28330499 PMC5363043

[23] Thomeer MJ Vansteenkiste J Verbeken EK Demedts M. Interstitial lung diseases: characteristics at diagnosis and mortality risk assessment. Respir Med. (2004) 98:567–73. 10.1016/j.rmed.2003.10.015 15191043

[24] Basu S Zhuang H Torigian DA Rosenbaum J Chen W Alavi A. Functional imaging of inflammatory diseases using nuclear medicine techniques. Semin Nucl Med. (2009) 39:124–45. 10.1053/j.semnuclmed.2008.10.006 19187805

[25] Hansell DM Goldin JG King TE Jr Lynch DA Richeldi L Wells AU. CT staging and monitoring of fibrotic interstitial lung diseases in clinical practice and treatment trials: a position paper from the Fleischner Society. Lancet Respir Med. (2015) 3:483–96. 10.1016/S2213-2600(15)00096-X 25975761

[26] Goldin J Elashoff R Kim HJ Yan X Lynch D Strollo D. Treatment of scleroderma-interstitial lung disease with cyclophosphamide is associated with less progressive fibrosis on serial thoracic high-resolution CT scan than placebo: findings from the scleroderma lung study. Chest. (2009) 136:1333–40. 10.1378/chest.09-0108 19892673 PMC2773360

[27] Win T Lambrou T Hutton BF Kayani I Screaton NJ Porter JC. ^18^F-Fluorodeoxyglucose positron emission tomography pulmonary imaging in idiopathic pulmonary fibrosis is reproducible: implications for future clinical trials. Eur J Nucl Med Mol Imaging. (2012) 39:521–8. 10.1007/s00259-011-1986-7 22258710

[28] Nobashi T Kubo T Nakamoto Y Handa T Koyasu S Ishimori T. ^18^F-FDG uptake in less affected lung field provides prognostic stratification in patients with interstitial lung disease. J Nucl Med. (2016) 57:1899–904. 10.2967/jnumed.116.174946 27339874

[29] Bellando-Randone S Tartarelli L Cavigli E Tofani L Bruni C Lepri G. ^18^F-fluorodeoxyglucose positron-emission tomography/CT and lung involvement in systemic sclerosis. Ann Rheum Dis. (2018) 78:577–8. 10.1136/annrheumdis-2018-eular.3748 30337426

[30] Bondue B Castiaux A Van Simaeys G Mathey C Sherer F Egrise D. Absence of early metabolic response assessed by ^18^F-FDG PET/CT after initiation of antifibrotic drugs in IPF patients. Respir Res. (2019) 20:10. 10.1186/s12931-019-0974-5 30646908 PMC6334423

[31] Desogere P Tapias LF Hariri LP Rotile NJ Rietz TA Probst CK. Type I collagen-targeted PET probe for pulmonary fibrosis detection and staging in preclinical models. Sci Transl Med. (2017) 9:eaaf4696. 10.1126/scitranslmed.aaf4696 28381537 PMC5568793

[32] Golestani R Razavian M Ye Y Zhang J Jung JJ Toczek J. Matrix metalloproteinase-targeted imaging of lung inflammation and remodeling. J Nucl Med. (2017) 58:138–43. 10.2967/jnumed.116.176198 27469361 PMC5209638

[33] Schniering J Benesova M Brunner M Haller S Cohrs S Frauenfelder T. Visualisation of interstitial lung disease by molecular imaging of integrin α_*v*_β_3_ and somatostatin receptor 2. Ann Rheum Dis. (2019) 78:218–27. 10.1136/annrheumdis-2018-214322 30448769

[34] Schniering J Borgna F Siwowska K Benesova M Cohrs S Hasler R. *In vivo* labeling of plasma proteins for imaging of enhanced vascular permeability in the lungs. Mol Pharm. (2018) 15:4995–5004. 10.1021/acs.molpharmaceut.8b00606 30265552

[35] Schniering J Guo L Brunner M Schibli R Ye S Distler O. Evaluation of ^99m^Tc-rhAnnexin V-128 SPECT/CT as a diagnostic tool for early stages of interstitial lung disease associated with systemic sclerosis. Arthritis Res Ther. (2018) 20:183. 10.1186/s13075-018-1681-1 30115119 PMC6097327

[36] Withana NP Ma X McGuire HM Verdoes M van der Linden WA Ofori LO. Non-invasive imaging of idiopathic pulmonary fibrosis using cathepsin protease probes. Sci Rep. (2016) 6:19755. 10.1038/srep19755 26797565 PMC4726431

[37] Han W Zaynagetdinov R Yull FE Polosukhin VV Gleaves LA Tanjore H. Molecular imaging of folate receptor beta-positive macrophages during acute lung inflammation. Am J Respir Cell Mol Biol. (2015) 53:50–9. 10.1165/rcmb.2014-0289OC 25375039 PMC4566110

[38] Paulos CM Turk MJ Breur GJ Low PS. Folate receptor-mediated targeting of therapeutic and imaging agents to activated macrophages in rheumatoid arthritis. Adv Drug Deliv Rev. (2004) 56:1205–17. 10.1016/j.addr.2004.01.012 15094216

[39] Low PS Kularatne SA. Folate-targeted therapeutic and imaging agents for cancer. Curr Opin Chem Biol. (2009) 13:256–62. 10.1016/j.cbpa.2009.03.022 19419901

[40] Nagai T Tanaka M Hasui K Shirahama H Kitajima S Yonezawa S. Effect of an immunotoxin to folate receptor beta on bleomycin-induced experimental pulmonary fibrosis. Clin Exp Immunol. (2010) 161:348–56. 10.1111/j.1365-2249.2010.04182.x 20550546 PMC2909418

[41] Silvola JMU Li XG Virta J Marjamaki P Liljenback H Hytonen JP. Aluminum fluoride-18 labeled folate enables *in vivo* detection of atherosclerotic plaque inflammation by positron emission tomography. Sci Rep. (2018) 8:9720. 10.1038/s41598-018-27618-4 29946129 PMC6018703

[42] Piscaer TM Müller C Mindt TL Lubberts E Verhaar JA Krenning EP. Imaging of activated macrophages in experimental osteoarthritis using folate-targeted animal single-photon-emission computed tomography/computed tomography. Arthritis Rheum. (2011) 63:1898–907. 10.1002/art.30363 21437875

[43] Jager NA Westra J Golestani R van Dam GM Low PS Tio RA. Folate receptor-beta imaging using ^99m^Tc-folate to explore distribution of polarized macrophage populations in human atherosclerotic plaque. J Nucl Med. (2014) 55:1945–51. 10.2967/jnumed.114.143180 25359878

[44] Chandrupatla D Jansen G Mantel E Low PS Matsuyama T Musters RP. Imaging and methotrexate response monitoring of systemic inflammation in arthritic rats employing the macrophage PET Tracer [^18^F]Fluoro-PEG-Folate. Contrast Media Mol Imaging. (2018) 2018:8092781. 10.1155/2018/8092781 29681783 PMC5841060

[45] Ayala-Lopez W Xia W Varghese B Low PS. Imaging of atherosclerosis in apoliprotein e knockout mice: targeting of a folate-conjugated radiopharmaceutical to activated macrophages. J Nucl Med. (2010) 51:768–74. 10.2967/jnumed.109.071324 20395331

[46] Winkel LC Groen HC van Thiel BS Müller C van der Steen AF Wentzel JJ. Folate receptor-targeted single-photon emission computed tomography/computed tomography to detect activated macrophages in atherosclerosis: can it distinguish vulnerable from stable atherosclerotic plaques? Mol Imaging. (2014) 13. 10.2310/7290.2013.00061 24757762

[47] Kelderhouse LE Robins MT Rosenbalm KE Hoylman EK Mahalingam S Low PS. Prediction of response to therapy for autoimmune/inflammatory diseases using an activated macrophage-targeted radioimaging agent. Mol Pharm. (2015) 12:3547–55. 10.1021/acs.molpharmaceut.5b00134 26333010

[48] Kraus VB McDaniel G Huebner JL Stabler TV Pieper CF Shipes SW. Direct in vivo evidence of activated macrophages in human osteoarthritis. Osteoarthr Cartilage. (2016) 24:1613–21. 10.1016/j.joca.2016.04.010 27084348 PMC4992586

[49] Papadopoulos V Baraldi M Guilarte TR Knudsen TB Lacapere JJ Lindemann P. Translocator protein (18kDa): new nomenclature for the peripheral-type benzodiazepine receptor based on its structure and molecular function. Trends Pharmacol Sci. (2006) 27:402–9. 10.1016/j.tips.2006.06.005 16822554

[50] Largeau B Dupont AC Guilloteau D Santiago-Ribeiro MJ Arlicot N. TSPO PET imaging: from microglial activation to peripheral sterile inflammatory diseases? Contrast Media Mol Imaging. (2017) 2017:6592139. 10.1155/2017/6592139 29114179 PMC5632884

[51] Canat X Guillaumont A Bouaboula M Poinot-Chazel C Derocq JM Carayon P. Peripheral benzodiazepine receptor modulation with phagocyte differentiation. Biochem Pharmacol. (1993) 46:551–4. 10.1016/0006-2952(93)90535-5 8394087

[52] Owen DR Yeo AJ Gunn RN Song K Wadsworth G Lewis A. An 18-kDa translocator protein (TSPO) polymorphism explains differences in binding affinity of the PET radioligand PBR28. J Cereb Blood Flow Metab. (2012) 32:1–5. 10.1038/jcbfm.2011.147 22008728 PMC3323305

[53] Owen DR Gunn RN Rabiner EA Bennacef I Fujita M Kreisl WC. Mixed-affinity binding in humans with 18-kDa translocator protein ligands. J Nucl Med. (2011) 52:24–32. 10.2967/jnumed.110.079459 21149489 PMC3161826

[54] Owen DR Howell OW Tang SP Wells LA Bennacef I Bergstrom M. Two binding sites for [^3^H]PBR28 in human brain: implications for TSPO PET imaging of neuroinflammation. J Cereb Blood Flow Metab. (2010) 30:1608–18. 10.1038/jcbfm.2010.63 20424634 PMC2949260

[55] Schiller HB Fernandez IE Burgstaller G Schaab C Scheltema RA Schwarzmayr T. Time- and compartment-resolved proteome profiling of the extracellular niche in lung injury and repair. Mol Syst Biol. (2015) 11:819. 10.15252/msb.20156123 26174933 PMC4547847

[56] Misharin AV Morales-Nebreda L Mutlu GM Budinger GR Perlman H. Flow cytometric analysis of macrophages and dendritic cell subsets in the mouse lung. Am J Respir Cell Mol Biol. (2013) 49:503–10. 10.1165/rcmb.2013-0086MA 23672262 PMC3824047

[57] Ayaub EA Dubey A Imani J Botelho F Kolb MRJ Richards CD. Overexpression of OSM and IL-6 impacts the polarization of pro-fibrotic macrophages and the development of bleomycin-induced lung fibrosis. Sci Rep. (2017) 7:13281. 10.1038/s41598-017-13511-z 29038604 PMC5643520

[58] Ashcroft T Simpson JM Timbrell V. Simple method of estimating severity of pulmonary fibrosis on a numerical scale. J Clin Pathol. (1988) 41:467–70. 10.1136/jcp.41.4.467 3366935 PMC1141479

[59] Seger S Stritt M Vezzali E Nayler O Hess P Groenen PMA. A fully automated image analysis method to quantify lung fibrosis in the bleomycin-induced rat model. PLoS ONE. (2018) 13:e0193057. 10.1371/journal.pone.0193057 29547661 PMC5856260

[60] Woessner JF Jr Boucek RJ. Connective tissue development in subcutaneously implanted polyvinyl sponge. I. Biochemical changes during development. Arch Biochem Biophys. (1961) 93:85–94. 10.1016/0003-9861(61)90319-8 13786178

[61] Betzel T Müller C Groehn V Müller A Reber J Fischer CR. Radiosynthesis and preclinical evaluation of 3′-Aza-2′-[^18^F]fluorofolic acid: a novel PET radiotracer for folate receptor targeting. Bioconjug Chem. (2013) 24:205–14. 10.1021/bc300483a 23273015

[62] Boss SD Müller C Siwowska K Schmid RM Groehn V Schibli R. Diastereomerically pure 6R- and 6S-3′-Aza-2′-^18^F-Fluoro-5-Methyltetrahydrofolates show unprecedentedly high uptake in folate receptor-positive KB tumors. J Nucl Med. (2019) 60:135–41. 10.2967/jnumed.118.213314 30042162

[63] Xia W Hilgenbrink AR Matteson EL Lockwood MB Cheng JX Low PS. A functional folate receptor is induced during macrophage activation and can be used to target drugs to activated macrophages. Blood. (2009) 113:438–46. 10.1182/blood-2008-04-150789 18952896

[64] van den Brule S Huaux F Uwambayinema F Ibouraadaten S Yakoub Y Palmai-Pallag M. Lung inflammation and thymic atrophy after bleomycin are controlled by the prostaglandin D2 receptor DP1. Am J Respir Cell Mol Biol. (2014) 50:212–22. 10.1165/rcmb.2012-0520OC 24003988

[65] Bellomo A Gentek R Bajenoff M Baratin M. Lymph node macrophages: scavengers, immune sentinels and trophic effectors. Cell Immunol. (2018) 330:168–74. 10.1016/j.cellimm.2018.01.010 29397903

[66] Junt T Moseman EA Iannacone M Massberg S Lang PA Boes M. Subcapsular sinus macrophages in lymph nodes clear lymph-borne viruses and present them to antiviral B cells. Nature. (2007) 450:110–4. 10.1038/nature06287 17934446

[67] Groves AM Win T Screaton NJ Berovic M Endozo R Booth H. Idiopathic pulmonary fibrosis and diffuse parenchymal lung disease: implications from initial experience with ^18^F-FDG PET/CT. J Nucl Med. (2009) 50:538–45. 10.2967/jnumed.108.057901 19289428

[68] Win T Screaton NJ Porter JC Ganeshan B Maher TM Fraioli F. Pulmonary ^18^F-FDG uptake helps refine current risk stratification in idiopathic pulmonary fibrosis (IPF). Eur J Nucl Med Mol Imaging. (2018) 45:806–15. 10.1007/s00259-017-3917-8 29335764 PMC5978900

[69] Win T Thomas BA Lambrou T Hutton BF Screaton NJ Porter JC. Areas of normal pulmonary parenchyma on HRCT exhibit increased FDG PET signal in IPF patients. Eur J Nucl Med Mol Imaging. (2014) 41:337–42. 10.1007/s00259-013-2514-8 23942907 PMC3890564

[70] Justet A Laurent-Bellue A Thabut G Dieudonne A Debray MP Borie R. [^18^F]FDG PET/CT predicts progression-free survival in patients with idiopathic pulmonary fibrosis. Respir Res. (2017) 18:74. 10.1186/s12931-017-0556-3 28449678 PMC5408423

[71] Motegi SI Fujiwara C Sekiguchi A Hara K Yamaguchi K Maeno T Clinical value of ^18^F-fluorodeoxyglucose positron emission tomography/computed tomography for interstitial lung disease and myositis in patients with dermatomyositis. J Dermatol. (2019) 46:213–8. 10.1111/1346-8138.14758 30614031

[72] Bondue B Sherer F Van Simaeys G Doumont G Egrise D Yakoub Y. PET/CT with ^18^F-FDG- and ^18^F-FBEM-labeled leukocytes for metabolic activity and leukocyte recruitment monitoring in a mouse model of pulmonary fibrosis. J Nucl Med. (2015) 56:127–32. 10.2967/jnumed.114.147421 25537989

[73] Khanna D Tashkin DP Denton CP Lubell MW Vazquez-Mateo C Wax S. Ongoing clinical trials and treatment options for patients with systemic sclerosis-associated interstitial lung disease. Rheumatology. (2018) 58:567–79. 10.1093/rheumatology/key151 29893938 PMC6434373

[74] Kolb M Bonella F Wollin L. Therapeutic targets in idiopathic pulmonary fibrosis. Respir Med. (2017) 131:49–57. 10.1016/j.rmed.2017.07.062 28947042

[75] Distler O Highland KB Gahlemann M Azuma A Fischer A Mayes MD. Nintedanib for systemic sclerosis-associated interstitial lung disease. N Engl J Med. (2019) 380:2518–28. 10.1056/NEJMoa1903076 31112379

[76] Huang J Maier C Zhang Y Soare A Dees C Beyer C. Nintedanib inhibits macrophage activation and ameliorates vascular and fibrotic manifestations in the Fra2 mouse model of systemic sclerosis. Ann Rheum Dis. (2017) 76:1941–8. 10.1136/annrheumdis-2016-210823 28814429

[77] Toda M Mizuguchi S Minamiyama Y Yamamoto-Oka H Aota T Kubo S. Pirfenidone suppresses polarization to M2 phenotype macrophages and the fibrogenic activity of rat lung fibroblasts. J Clin Biochem Nutr. (2018) 63:58–65. 10.3164/jcbn.17-111 30087545 PMC6064814

[78] Khanna D Denton CP Lin CJF van Laar JM Frech TM Anderson ME. Safety and efficacy of subcutaneous tocilizumab in systemic sclerosis: results from the open-label period of a phase II randomised controlled trial (faSScinate). Ann Rheum Dis. (2018) 77:212–20. 10.1136/annrheumdis-2017-211682 29066464 PMC5867414

[79] Byrne AJ Mathie SA Gregory LG Lloyd CM. Pulmonary macrophages: key players in the innate defence of the airways. Thorax. (2015) 70:1189–96. 10.1136/thoraxjnl-2015-207020 26286722

[80] Li H Nagai T Hasui K Matsuyama T. Depletion of folate receptor beta-expressing macrophages alleviates bleomycin-induced experimental skin fibrosis. Mod Rheumatol. (2014) 24:816–22. 10.3109/14397595.2013.879415 24498991

[81] Feng Y Shen J Streaker ED Lockwood M Zhu Z Low PS. A folate receptor beta-specific human monoclonal antibody recognizes activated macrophage of rheumatoid patients and mediates antibody-dependent cell-mediated cytotoxicity. Arthritis Res Ther. (2011) 13:R59. 10.1186/ar3312 21477314 PMC3132054

[82] Lynn RC Feng Y Schutsky K Poussin M Kalota A Dimitrov DS. High-affinity FRbeta-specific CAR T cells eradicate AML and normal myeloid lineage without HSC toxicity. Leukemia. (2016) 30:1355–64. 10.1038/leu.2016.35 26898190 PMC4889499

[83] Cao Z Lis R Ginsberg M Chavez D Shido K Rabbany SY. Targeting of the pulmonary capillary vascular niche promotes lung alveolar repair and ameliorates fibrosis. Nat Med. (2016) 22:154–62. 10.1038/nm.4035 26779814 PMC4872630

[84] Chen DL Cheriyan J Chilvers ER Choudhury G Coello C Connell M. Quantification of lung PET images: challenges and opportunities. J Nucl Med. (2017) 58:201–7. 10.2967/jnumed.116.184796 28082432 PMC5288738

[85] Guerra L Ponti E Morzenti S Spadavecchia C Crivellaro C. Respiratory motion management in PET/CT: applications and clinical usefulness. Curr Radiopharm. (2017) 10:85–92. 10.2174/1874471010666170519165918 28530533

[86] Reddy JA Xu LC Parker N Vetzel M Leamon CP Preclinical evaluation of ^99m^Tc-EC20 for imaging folate receptor-positive tumors. J Nucl Med. (2004) 45:857–66. 15136637

[87] Vande Velde G Poelmans J De Langhe E Hillen A Vanoirbeek J Himmelreich U. Longitudinal micro-CT provides biomarkers of lung disease that can be used to assess the effect of therapy in preclinical mouse models, and reveal compensatory changes in lung volume. Dis Models Mech. (2016) 9:91–8. 10.1242/dmm.020321 26563390 PMC4728330

[88] Aichler M Kunzke T Buck A Sun N Ackermann M Jonigk D. Molecular similarities and differences from human pulmonary fibrosis and corresponding mouse model: MALDI imaging mass spectrometry in comparative medicine. Lab Invest. (2018) 98:141–9. 10.1038/labinvest.2017.110 29035378

