# Supplementary material for: 18F-AzaFol for Detection of Folate Receptor-β Positive Macrophages in Experimental Interstitial Lung Disease—A Proof-of-Concept Study
Source: Front Immunol. 2019 Nov 22;10:2724. doi: 10.3389/fimmu.2019.02724 (PMC6883947; doi:10.3389/fimmu.2019.02724)
Supplement: Supplementary file 1 [file Data_Sheet_1.docx]

Supplementary Material

## Supplementary Figures

**
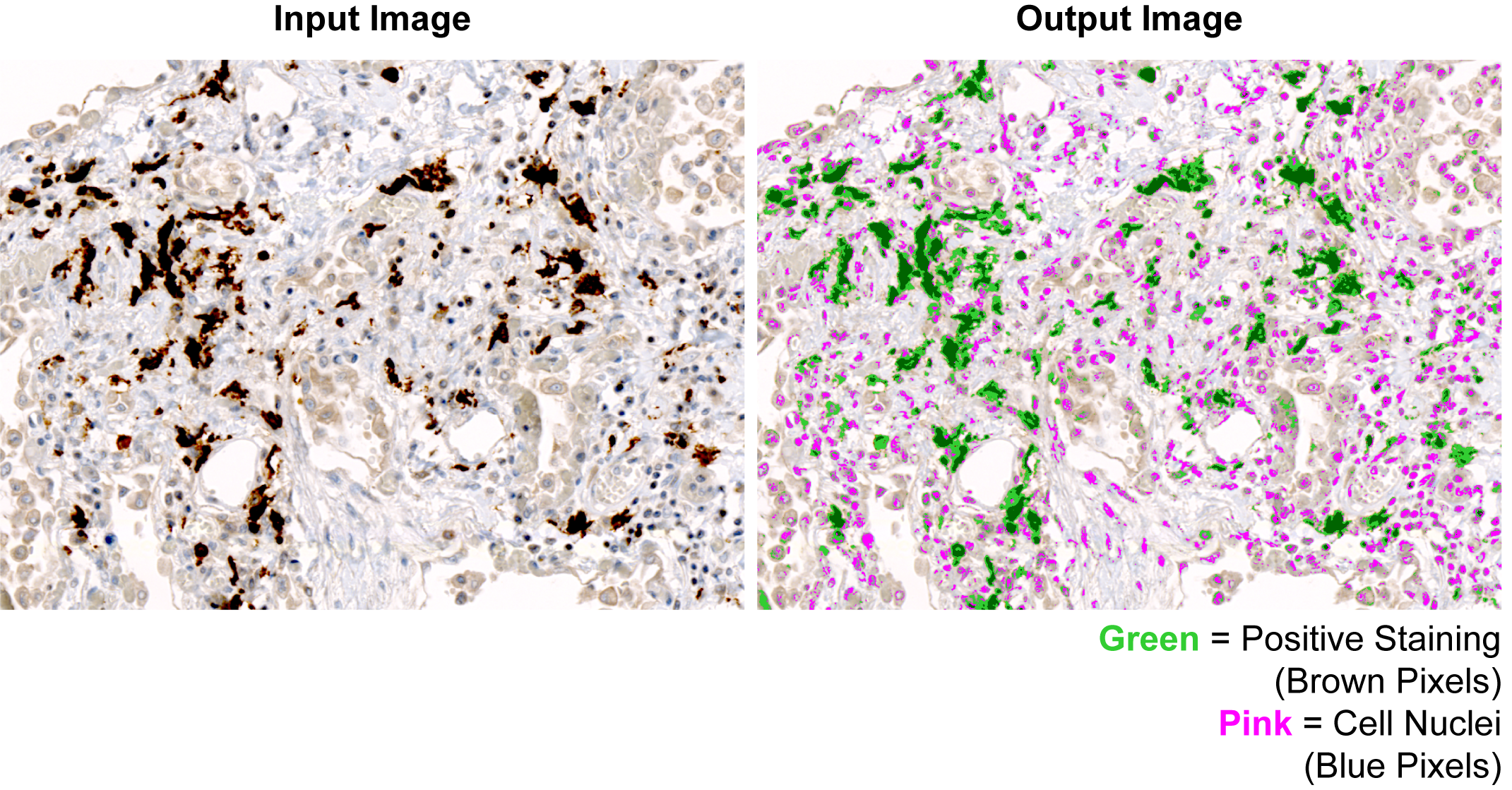
**

**Supplementary Figure 1.** **Representative example of the automated semi-quantification analysis of the immunohistochemical stainings.** For automated and objective (observer-unbiased) quantification of target expression in immunohistochemical stainings, an in-house developed *MATLAB*-based script was used quantifying the target-positive pixels and cell nuclei-positive pixels. In the output image, target-positive (=brown) pixels are false-colored in green, whereas cell-nuclei positive (=blue) pixels are false-colored in pink.


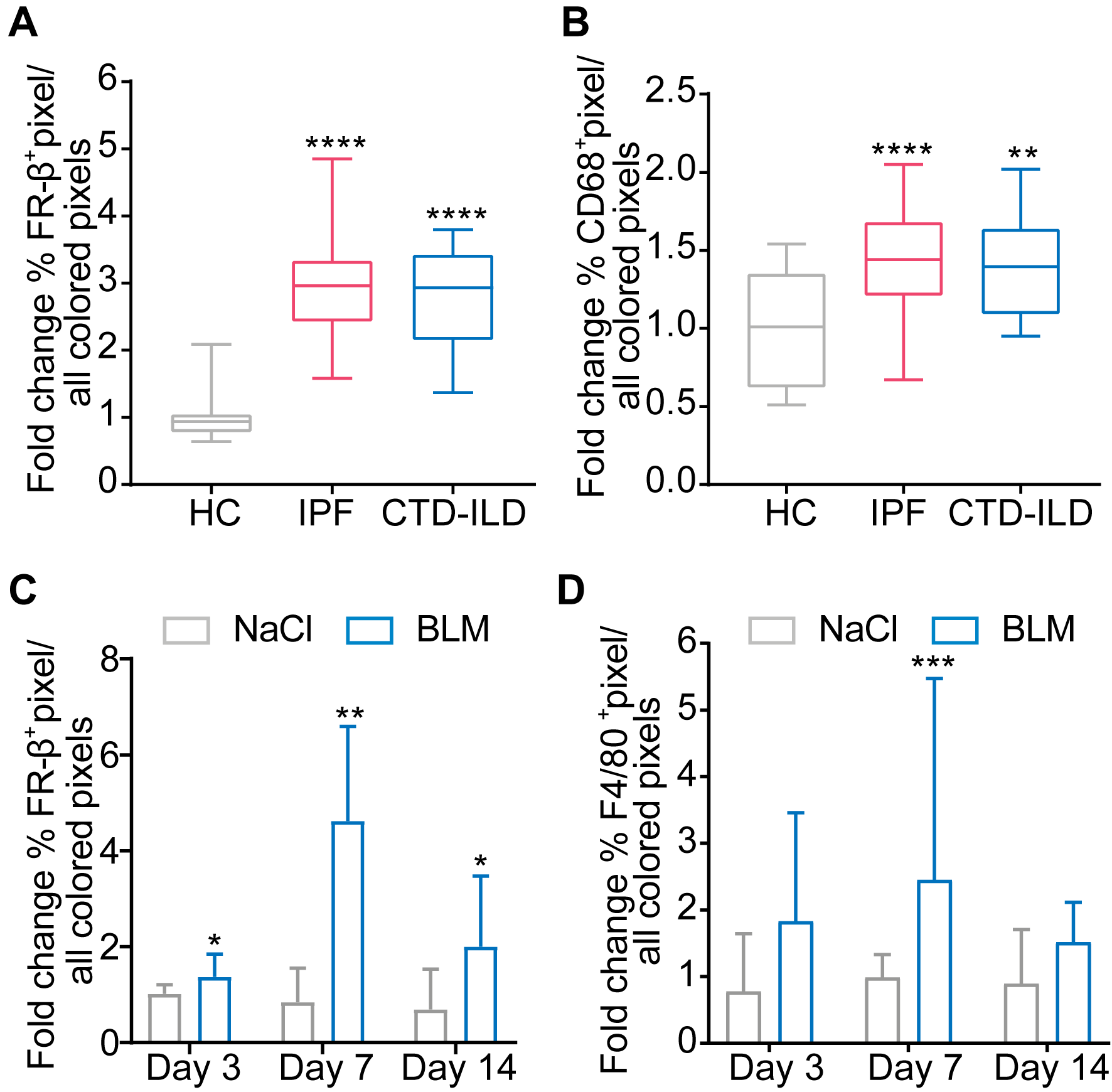


**Supplementary Figure 2.** **Automated semi-quantification of FR-β and CD68 or F4/80 expression in human and experimental ILD taking increased cell numbers and tissue consolidations into account.** (**A**) Fold change of the percentage of FR-β+ (brown) pixels in relation to all colored (brown + blue) pixels in patients with IPF and CTD-ILD compared to healthy controls. (**B**) Fold change of the percentage of CD68+ (brown) pixels in relation to all colored (brown + blue) pixels in patients with IPF and CTD-ILD compared to healthy controls. (**C**) Fold change of the percentage of FR-β+ (red) pixels in relation to all colored (red + blue) pixels in BLM-treated compared to NaCl-treated controls at days 3, 7 and 14. (**D**) Fold change of the percentage of F4/80+ (brown) pixels in relation to all colored (brown + blue) pixels in BLM-treated compared to NaCl-treated controls at days 3, 7 and 14. For (**A** to **B**) data are displayed as box plots with min/max values. For statistical analysis, the Kruskal-Wallis test with Dunn’s multiple correction was applied. For (**C** to **D**) data are presented as medians ± IQR. For statistical analysis, the Mann-Whitney U test was applied (*p<0.05, **p<0.01, ***p<0.001, ****p<0.0001).


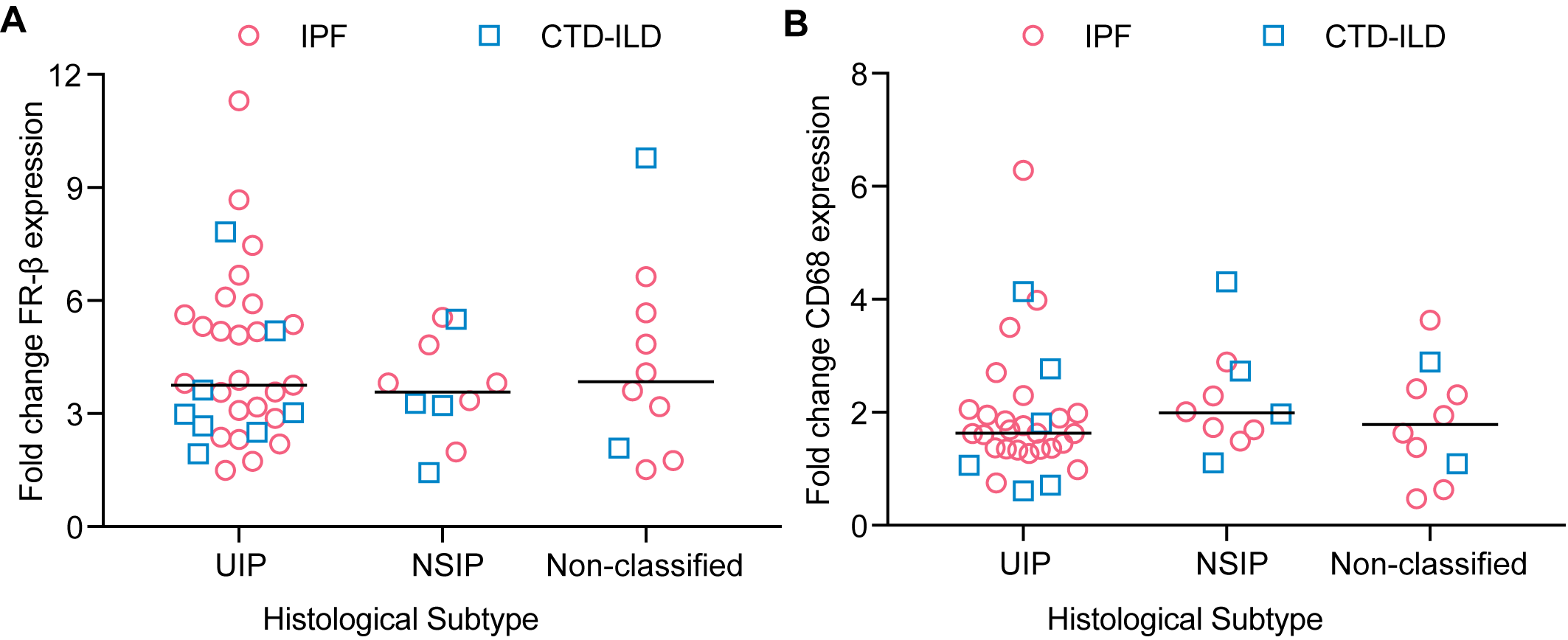


.

**Supplementary Figure 3.** **Upregulation of FR-β and CD68 expression is independent of the histological subtype.** (**A**) FR-β expression dependent on the histological subtype. (**B**) CD68 expression dependent on the histological subtype. For (**A** and **B**) individual data points for each patient are plotted with the black line indicating the grand median.


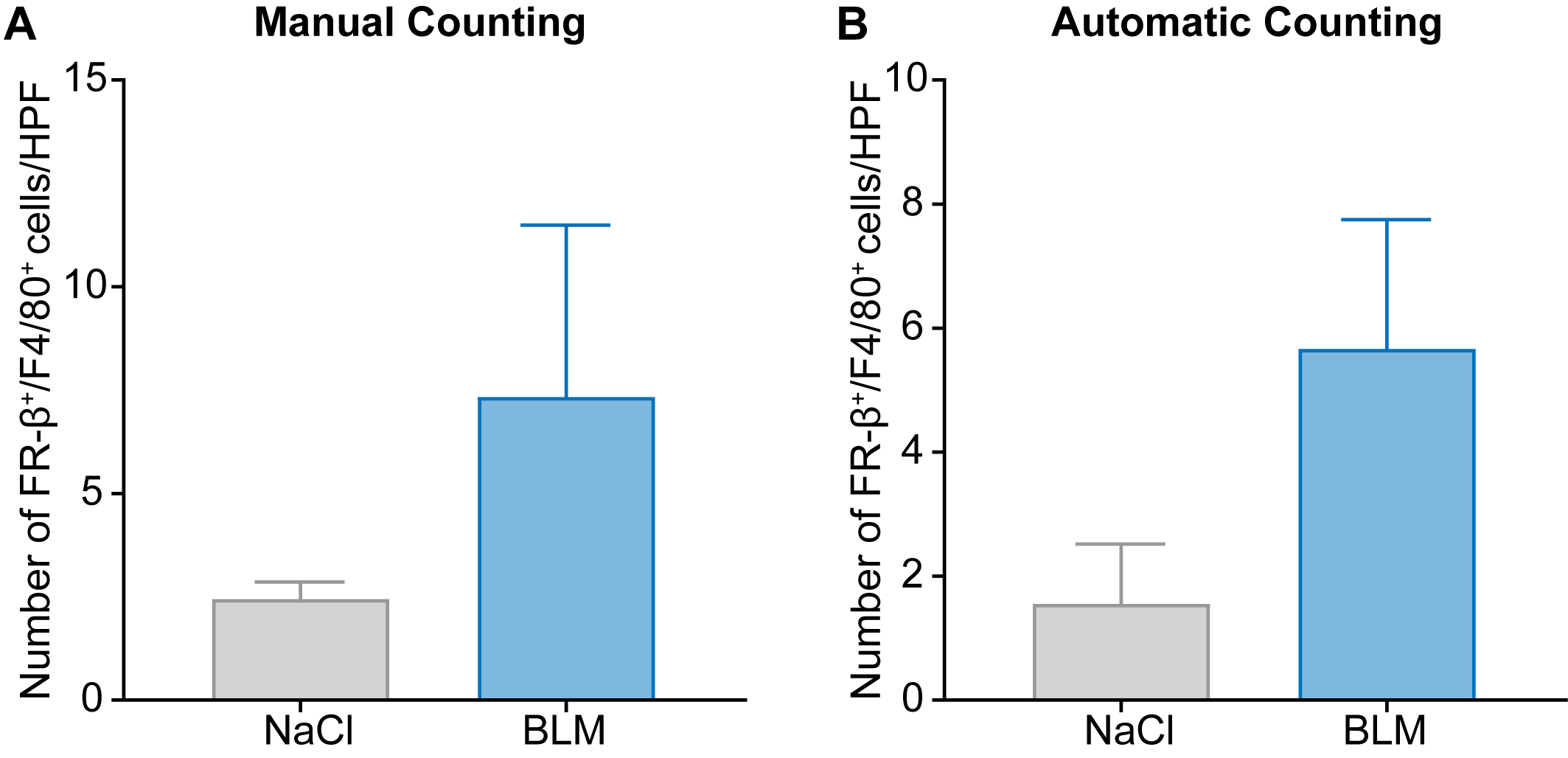


**Supplementary Figure 4. Manual and automated semi-quantification of FR-β/F4/80 double positive cells.** (**A**) Manual counting was performed by two blinded examiners. (**B**) Automated counting was performed using Orbit image analysis software (Modules: Object Detection and Object Classification). Both manual and automated counting was carried out on representative HPFs (630x magnification) extracted from immunofluorescent double stainings from three NaCl controls and three BLM-treated animals at Day 7. Data are expressed as means ± S.D.


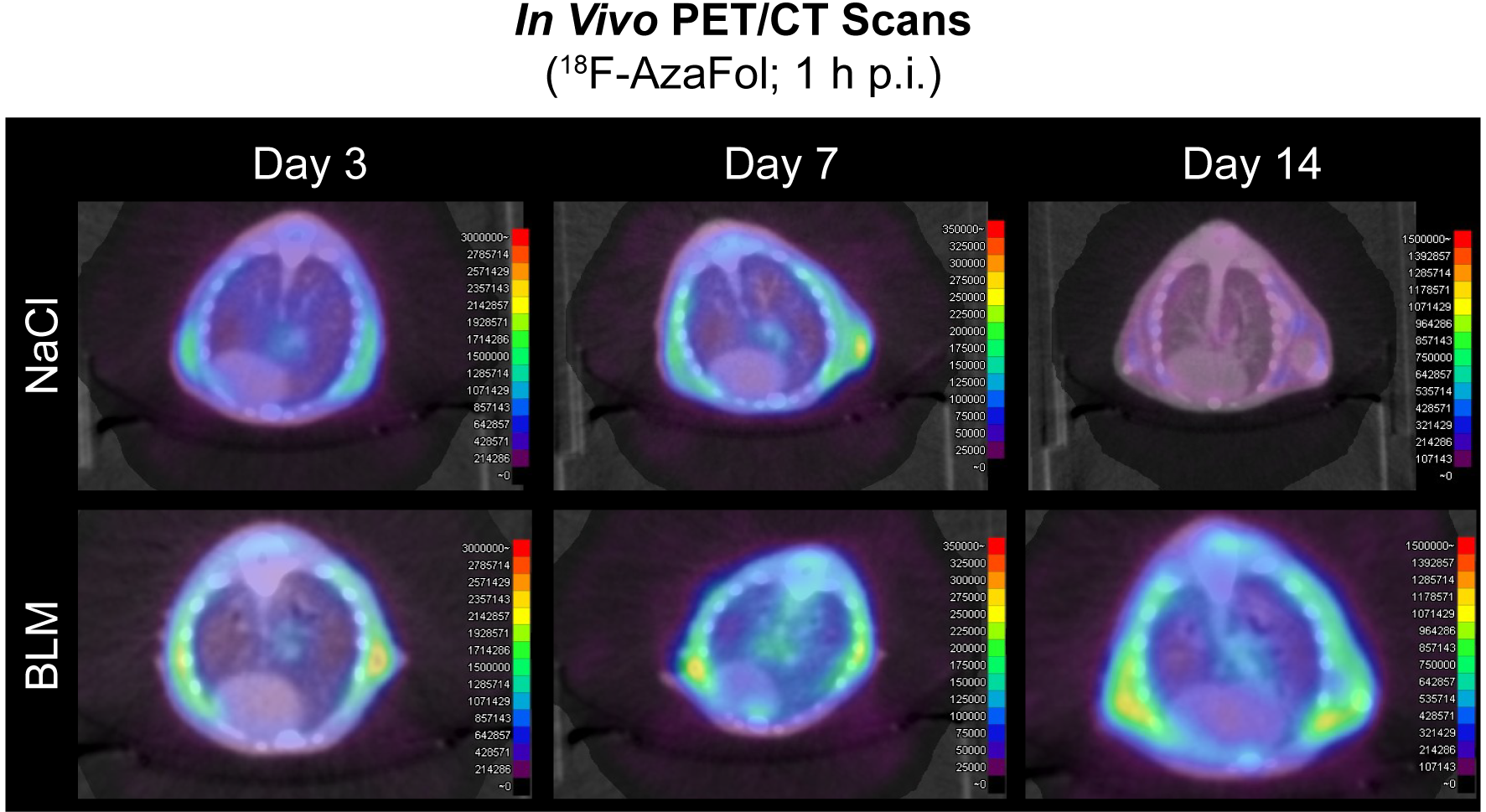


**Supplementary Figure 5. *In vivo* chest imaging with ^18^F-AzaFol-PET/CT in experimental ILD.** *In vivo* PET/CT scans restricted to the chest region of saline controls and BLM-treated mice at days 3, 7, and 14 that were performed 1 h after injection of ^18^F-AzaFol. Transaxial projections of the lung windows are shown. Scales were standardized for each time point separately between NaCl and BLM-treated mice.

## Supplementary Tables

**Supplementary Table 1. Patients’ demographics and clinical characteristics.** Data are presented as absolute values (*n*), percentages (%) or medians (Q1, Q3).

| **Variable** | **IPF** | **CTD-ILD^*^** |
| --- | --- | --- |
| **Number of patients** | 39 | 14 |
| **Age (years)** | 61 (53, 66) | 57.5 (47.5, 62) |
| **Sex *n* (%)**  Female  Male | 11 (28%)  28 (72%) | 5 (36%)  9 (64%) |
| **Ethnicity *n* (%)**  Caucasian  Asian | 39 (100%)  0 (0%) | 13 (93%)  1 (7%) |
| **Ever smoking *n* (%)** | 13 (33%) | 7 (50%) |
| **Histological subtype**  UIP  NSIP  Non-classified | 25 (64%)  6 (15%)  8 (21%) | 8 (57%)  4 (29%)  2 (14%) |
| **Disease duration (years)** | 5 (4, 10) | 5 (3, 8) |
| **FVC (%)** | 50 (37, 57) | 47 (39, 72) |
| **FEV1 (%)** | 55 (38.5, 63.5) | 46 (40, 65) |
| **TLC (%)** | 49.5 (38.3, 55) | 50 (41, 63) |
| **DLCO (%)** | 28 (21, 40) | 28 (20, 32) |
| **mPAP** ^†^ **(mmHg)** | 33 (32.5, 38) | 30 (30, 38.3) |
| **Concomitant P(A)H *n (*%)** | 5 (13%) | 6 (43%) |
| **P(A)H Treatment *n* (%)** | 4 (10%) | 4 (29%) |
| **Immunosuppression**^‡^ ***n* (%)** | 28 (72%) | 12 (86%) |

^*^ILD associated with systemic sclerosis (n=5), rheumatoid arthritis (n=4), antisynthetase syndrome (n=1), others (n=4);

^†^ diagnosed by right heart catherization;

^‡^ Prednisone, azathioprin, cyclophosphamide, mycophenolate mofetil, tacrolimus, pirfenidon, rituximab, cyclosporin A, leflunomide, remicade, tocilizumab, imatinib

Supplementary Table 2. Biodistribution data expressed as percentage of injected activity per gram of tissue (% IA/g) of ^18^F-AzaFol (1 h p.i.) in saline-treated controls and BLM-treated mice at days 3, 7 and 14 after BLM instillation. Data are expressed as mean ± S.D. For all experiments: n=3-4 for saline-treated mice, n=3-4 for BLM-treated mice and n=2-4 for BLM-treated mice receiving receptor blockade.

|  |  | | | | | | | | |
| --- | --- | --- | --- | --- | --- | --- | --- | --- | --- |
|  |  | **Day 3** |  |  | **Day 7** |  |  | **Day 14** |  |
| Organ | **NaCl** | **BLM** | **BLM + Block** | **NaCl** | **BLM** | **BLM + Block** | **NaCl** | **BLM** | **BLM + Block** |
|  | [% IA/g] | | | [% IA/g] | | | [% IA/g] | | |
| Blood | 0.60 ± 0.22 | 1.09 ± 0.17 | 0.97 ± 0.23 | 0.75 ± 0.37 | 1.23 ± 0.75 | 0.97 ± 0.45 | 0.88 ± 0.08 | 0.96 ± 0.14 | 0.77 ± 0.19 |
| Heart | 0.66 ± 0.24 | 1.19 ± 0.25 | 0.48 ± 0.12 | 1.08 ± 0.32 | 1.41 ± 0.66 | 0.56 ± 0.21 | 1.11 ± 0.21 | 1.32 ± 0.27 | 0.48 ± 0.14 |
| Lung | 1.12 ± 0.32 | 2.70 ± 0.66 | 1.15 ± 0.24 | 1.78 ± 0.15 | 3.33 ± 0.77 | 1.62 ± 0.84 | 1.74 ± 0.29 | 3.40 ± 1.28 | 0.77 ± 0.11 |
| Thymus | 0.81 ± 0.45 | 1.66 ± 0.29 | 0.44 ± 0.05 | 1.91 ± 0.53 | 3.06 ± 0.74 | 0.86 ± 0.43 | 1.70 ± 0.50 | 3.03 ± 1.34 | 0.60 ± 0.08 |
| Spleen | 0.50 ± 0.18 | 0.93 ± 0.06 | 0.35 ± 0.05 | 0.82 ± 0.27 | 1.07 ± 0.37 | 0.44 ± 0.13 | 0.89 ± 0.15 | 0.92 ± 0.07 | 0.39 ± 0.09 |
| Intestine | 1.02 ± 0.48 | 1.74 ± 0.05 | 1.24 ± 0.86 | 1.47 ± 1.01 | 2.69 ± 1.26 | 2.23 ± 0.37 | 1.68 ± 0.34 | 2.03 ± 0.42 | 2.05 ± 0.58 |
| Liver | 4.64 ± 2.07 | 8.44 ± 1.35 | 6.93 ± 0.84 | 5.74 ± 1.19 | 8.91 ± 3.39 | 3.74 ± 2.42 | 5.97 ± 1.21 | 7.30 ± 0.73 | 4.37 ± 1.06 |
| Muscle | 0.87 ± 0.06 | 1.25 ± 0.37 | 0.31 ± 0.11 | 0.80 ± 0.18 | 0.94 ± 0.29 | 0.37 ± 0.17 | 1.17 ± 0.51 | 1.16 ± 0.17 | 0.39 ± 0.03 |
| Bone | 0.87 ± 0.31 | 1.34 ± 0.25 | 0.50 ± 0.17 | 1.36 ± 0.50 | 1.62 ± 0.67 | 0.58 ± 0.32 | 1.37 ± 0.19 | 1.65 ± 0.19 | 0.79 ± 0.26 |
| Lymph nodes | 4.74 ± 1.46 | 8.50 ± 3.17 | 1.42 ± 1.30 | 4.74 ± 1.14 | 6.42 ± 1.98 | 1.49 ± 0.57 | 4.36 ± 3.10 | 8.87 ± 4.86 | 1.70 ± 0.85 |

**Supplementary Table 3. Biodistribution data expressed as percentage of injected activity per organ (% IA/organ) of ^18^F-AzaFol (1 h p.i.) in saline-treated controls and BLM-treated mice at days 3, 7 and 14 after BLM instillation.** Data are expressed as mean ± S.D. For all experiments: n=3-4 for saline-treated mice, n=3-4 for BLM-treated mice and n=2-4 for BLM-treated mice receiving receptor blockade.

|  |  | Day 3 |  |  | Day 7 |  |  | Day 14 |  |
| --- | --- | --- | --- | --- | --- | --- | --- | --- | --- |
| Organ | **NaCl** | **BLM** | **BLM + Block** | **NaCl** | **BLM** | **BLM + Block** | **NaCl** | **BLM** | **BLM + Block** |
|  | [% IA/organ] | | | [% IA/organ] | | | [% IA/organ] | | |
| Heart | 0.30 ± 0.42 | 0.11 ± 0.02 | 0.05 ± 0.01 | 0.12 ± 0.04 | 0.13 ± 0.07 | 0.05 ± 0.02 | 0.12 ± 0.02 | 0.13 ± 0.04 | 0.05 ± 0.01 |
| Lung | 0.22 ± 0.06 | 0.63 ± 0.14 | 0.29 ± 0.07 | 0.31 ± 0.06 | 1.01 ± 0.29 | 0.48 ± 0.21 | 0.34 ± 0.06 | 0.72 ± 0.07 | 0.24 ± 0.07 |
| Thymus | 0.04 ± 0.02 | 0.10 ± 0.02 | 0.02 ± 0.01 | 0.09 ± 0.01 | 0.12 ± 0.03 | 0.04 ± 0.02 | 0.08 ± 0.03 | 0.11 ± 0.02 | 0.03 ± 0.01 |
| Spleen | 0.04 ± 0.01 | 0.07 ± 0.01 | 0.02 ± 0.01 | 0.05 ± 0.02 | 0.07 ± 0.01 | 0.03 ± 0.01 | 0.06 ± 0.01 | 0.06 ± 0.01 | 0.03 ± 0.01 |
